# Temporal profile of intracranial pressure and cerebrovascular reactivity in severe traumatic brain injury and association with fatal outcome: An observational study

**DOI:** 10.1371/journal.pmed.1002353

**Published:** 2017-07-25

**Authors:** Hadie Adams, Joseph Donnelly, Marek Czosnyka, Angelos G. Kolias, Adel Helmy, David K. Menon, Peter Smielewski, Peter J. Hutchinson

**Affiliations:** 1 Division of Neurosurgery, Department of Clinical Neuroscience, Addenbrooke’s Hospital, University of Cambridge, Cambridge, United Kingdom; 2 Institute of Electronic Systems, Warsaw University of Technology, Warsaw, Poland; 3 Department of Anaesthesia, Addenbrooke’s Hospital, University of Cambridge, Cambridge, United Kingdom; Oregon Health and Science University, UNITED STATES

## Abstract

**Background:**

Both intracranial pressure (ICP) and the cerebrovascular pressure reactivity represent the dysregulation of pathways directly involved in traumatic brain injury (TBI) pathogenesis and have been used to inform clinical management. However, how these parameters evolve over time following injury and whether this evolution has any prognostic importance have not been studied.

**Methods and findings:**

We analysed the temporal profile of ICP and pressure reactivity index (PRx), examined their relation to TBI-specific mortality, and determined if the prognostic relevance of these parameters was affected by their temporal profile using mixed models for repeated measures of ICP and PRx for the first 240 hours from the time of injury. A total of 601 adults with TBI, admitted between September 2002 to January 2016, and with high-resolution continuous monitoring from a single centre, were studied. At 6 months postinjury, 133 (19%) patients had a fatal outcome; of those, 88 (78%) died from nonsurvivable TBI or brain death. The difference in mean ICP between those with a fatal outcome and functional survivors was only significant for the first 168 hours after injury (all *p* < 0.05). For PRx, those patients with a fatal outcome also had a higher (more impaired) PRx throughout the first 120 hours after injury (all *p* < 0.05). The separation of ICP and PRx was greatest in the first 72 hours after injury. Mixed models demonstrated that the explanatory power of the PRx decreases over time; therefore, the prognostic weight assigned to PRx should similarly decrease. However, the ability of ICP to predict a fatal outcome remained relatively stable over time. As control of ICP is the central purpose of TBI management, it is likely that some of the information that is reflected in the natural history of ICP changes is no longer apparent because of therapeutic intervention.

**Conclusions:**

We demonstrated the temporal evolution of ICP and PRx and their relationship with fatal outcome, indicating a potential early prognostic and therapeutic window. The combination of dynamic monitoring variables and their time profile improved prediction of outcome. Therefore, time-driven dynamic modelling of outcome in patients with severe TBI may allow for more accurate and clinically useful prediction models. Further research is needed to confirm and expand on these findings.

## Abstract 2

Using continuous monitoring data of patients with traumatic brain injury, Hadie Adams and colleagues examine the associations of temporal patterns of intracranial pressure and cerebrovascular reactivity with death over 10 days postinjury.

## Author summary

**Why was this study done?:**

- Following a head injury, one of the most damaging aspects of brain trauma is an elevated pressure inside the skull due to brain swelling.
- Knowledge of the relationship between the pressure inside the skull and time following an injury could assist in capturing and potentially treating the most critical episodes and could also identify the patients who might be at risk of poor outcome.

**What did the researchers do and find?:**

- We investigated the relation of the pressure inside the skull and the brain's ability to regulate its blood flow with time over the first 10 days following injury.
- Data from the first 3 days following injury seem to be able to predict death better than data from the other days.

**What do these findings mean?:**

- There might be a potential early window to treat patients and predict who was fatally injured following their head injury.
- When developing models to predict death following brain trauma, it seems to be important to take into account when the brain physiology data was captured.

## Introduction

Traumatic brain injury (TBI) is a major worldwide cause of morbidity and mortality [1]; in Europe alone, some 7.7 million people are living with TBI-induced disabilities [2], and of those with severe TBI (sTBI), a quarter to a third will die [3]. Furthermore, rates of severe morbidity and mortality have not improved over the last 20 years [1,3]. This burden of disability and mortality highlights the urgent need for novel strategies to decrease the prevalence and improve the management of TBI [4].

Because the injured brain is vulnerable to metabolic, haemodynamic, and pressure-induced insults [5–8], the overarching principle of the acute phase management focusses on the early detection, prevention, and effective treatment of these secondary injuries [9]. Intracranial hypertension and deranged cerebral haemodynamic regulation are key secondary injury mechanisms and have been the subject of intensive research since the introduction of clinical intracranial pressure (ICP) monitoring in the second half of the 20th century [10,11]. While ICP monitoring remains a key element of therapeutic strategies and an objective standard for measuring and monitoring sTBI patients, recent evidence has called into question how we should be using, interpreting, or acting on ICP [12,13]. Additionally, the recently published Randomized Evaluation of Surgery with Craniectomy for Uncontrollable Elevation of Intracranial Pressure (RESCUEicp) trial found that although the ICP-lowering strategy of decompressive craniectomy (DC) decreases mortality, it also increases the likelihood of the patient living with serious functional impairments [14].

Monitoring of cerebral haemodynamic regulation has also been used as a method for identifying vulnerable patients and informing patient management. Although early methods of cerebral vascular regulation required cerebral blood flow (CBF) measurements before and after a vasopressor challenge to give a snapshot of cerebrovascular function [15,16], modern methods such as the pressure reactivity index (PRx) assess cerebral haemodynamic regulation from spontaneous fluctuations of arterial blood pressure (ABP) and ICP and allow continuous cerebrovascular pressure reactivity monitoring [17,18]. Cerebrovascular reactivity reflects processes that maintain CBF at metabolically appropriate levels, and PRx has been associated with patient outcome in several studies [19–21]. In addition, PRx has been proposed as a potential guide for cerebral perfusion pressure (CPP) management [22,23]. To our knowledge, no study has shown that monitoring of the cerebral pressure reactivity index has any effect on outcome.

Taking together recent evidence concerning ICP and the paucity of clinical effectiveness data concerning PRx monitoring, we must first understand the temporal patterns, clinical sequelae, and relevance of secondary injuries before we can successfully integrate neuromonitoring into personalised patient management. In particular, the natural history of both ICP and PRx following sTBI is not well characterised; while some studies have investigated how ICP changes over the monitoring period [24–28], these studies have not taken into account the crucial reference time point—the precise time of acquiring the injury. Accurate elucidation of temporal patterns in ICP and PRx is essential from both clinical and research perspectives. Clinically, knowledge of temporal patterns in neuromonitoring data could assist in the decision process of how long to record ICP in order to capture and potentially treat the most critical episodes and also to allow early identification of patients with a severe injury who might be at risk of death or persisting neurologic disability or require neuroprotective therapies or early surgery. From a research perspective, temporal patterns of neuromonitoring data may be useful for studying the response to new interventions, studying biomarkers associated with ICP or PRx disturbance, or developing prognostic models.

In this prospectively collected neuromonitoring cohort in sTBI patients, we retrospectively analysed the temporal profile of ICP and PRx, examined their relation to TBI-specific mortality, and determined if the prognostic relevance of these parameters was affected by their temporal profile. On the basis of these analyses, we assessed a time-driven dynamic outcome prediction model based on continuously monitored ICP and PRx.

## Methods

### Study population

Data collection was approved by the relevant research ethics committee (30 REC 97/291) and also includes routine clinical data not collected under patient/next-of-kin consent/assent that was anonymized in accordance with United Kingdom legislation. As part of a prospective observational neuro-monitoring cohort study, we included adults (≥16 years) who had sustained a TBI and required management with ventilation and ICP monitoring at the Neuro Critical Care Unit (NCCU) at Addenbrooke’s Hospital–Cambridge University Hospital NHS Foundation Trust. Patients were admitted during a period from 1 September 2002 to 31 January 2016 and were managed according to TBI guidelines as part of tiered therapeutic protocols that aim to control raised ICP and ensure adequate CPP. The management of patients with severe TBI at the neurocritical care unit at Addenbrooke’s Hospital is based on the principles outlined in 1999 [9]. ICP/CPP management protocols are not static but are updated on the basis of available evidence/expert consensus. Three versions of the study protocol encompassed the 13-year observation period for this study. The differences between these protocols can be summarized as follows: the upper CPP target was changed from 70 to 60 mm Hg in 2003 on the basis of the paper by Robertson et al. [29], and the lower limit of the end tidal CO2 target was changed from 4 kPa to 4.5 kPa on the basis of Coles et al. [30]. In the latest version of the protocol (after the enrolment of the last patient in this study), the ICP target was relaxed to 25 mm Hg. The latest version of our institutional ICP/CPP management protocol is publicly available: http://cambridgecriticalcare.net/nccu-tbi-protocol/ (the management protocol has been attached in S1 Supporting Information).

The computerised data storage protocol was reviewed and approved by the local ethics committee of Addenbrooke’s Hospital, Cambridge University, and the NCCU User’s Group. The study results are reported in accordance with the Transparent Reporting of a multivariable prediction model for Individual Prognosis Or Diagnosis (TRIPOD) statement (S1 Appendix) [31].

### Data elements and outcome measures

Clinical data were abstracted and cross-validated from the emergency medical service reports, hospital records, surgical reports, and medical imaging using standardised definitions derived by group consensus prior to collection of the data. Demographics and other baseline characteristics including date and time of injury and best preintubation score on the Glasgow Coma Scale (GCS) were determined from the emergency medical service reports and the patient neurocritical care admission assessment. Primary injury classification (diffuse injury or mass lesion) was determined on the basis of the Marshall classification of the initial CT image of the head. If the initial CT image of the head was not available, the classification was done on the basis of the of the next available CT image of the head. Surgical interventions were further classified as to whether a craniotomy for a mass lesion or a primary or secondary DC had occurred. Primary DC was defined as a DC early in the management, with the patient undergoing emergent surgery (for example, for EDH or SDH evacuation) and the bone flap left out following the initial surgery. Secondary DC was defined as an adjunct for persistent intracranial hypertension when other ICP measures fail.

### Patient outcome

Since non-neurologic organ dysfunction can significantly impact mortality following sTBI, it is important to distinguish between fatality due to neurological causes (nonsurvivable TBI or brain death) and fatality due to a non-neurologic cause [32–34]. The cause of death and contributing factors to mortality were determined by review of hospital records or by the acquisition of a death certificate or autopsy reports.

In order to assess the prognostic and predictive ability of brain physiological parameters for mortality, the main outcome measure for the current study was mortality at 6 months postinjury due to a neurological cause. The latter group was compared to functional survivors; this has been defined as survivors with severe or moderate disability or good recovery. The time course of ICP and PRx was also further stratified by functional status at 6 months postinjury using the Glasgow Outcome Scale (GOS) to evaluate if this TBI cohort generally reflects the same functional outcome distribution commonly reported in severe TBI cohorts and if our results could be generalised. GOS was determined by clinical research nurses in 3 ways: (1) through the hospital record, if the patient died in-hospital; (2) during a hospital visit to the neurotrauma or neurorehabilitation clinic 6 months after admission; or (3) by phone interview, either of the patient himself/herself or his/her relatives [35].

### Data acquisition and analyses

ICP was monitored with an intraparenchymal microsensor inserted into the frontal lobe (Codman ICP MicroSensor, Codman & Shurtleff, Raynham, Massachusetts), and ABP was monitored in the radial or femoral artery with a 0 calibration at the level of the right atrium (Baxter Healthcare, California, United States; Sidcup, UK). Data were sampled at a minimum of 100 Hz with proprietary data acquisition software (ICM+, Cambridge Enterprise, Cambridge, UK, http://www.neurosurg.cam.ac.uk/icmplus) and stored for subsequent analysis.

PRx was calculated as the Pearson correlation of 30 consecutive 10-second average values of ABP and ICP. A 10-second average was used to reduce the influence of respiratory and pulse waveforms. A 300-second moving window, updated every 1 minute, was used to generate continuous PRx values.

Mean values of physiologic variables were calculated in 24-hour epochs referenced from the time of injury (T_0_) to 240 hours postinjury (T_240_). All mean ICP or PRx values for every 24-hour epoch of every study participant were only calculated if 50% or more of the epoch’s recording was available. Recording data of all patients were reviewed for artefacts, and if present, those minutes were excluded.

Because of the latency between the time of injury and the start of the recording (e.g., due to interhospital transfers, delayed ICP transducer insertion, and/or delay in connecting to data acquisition software), very early time points are frequently missing. Other reasons why gaps could occur in the recordings are as follows: the patient required a shorter period of monitoring than 240 hours postinjury (i.e., ICP within normal ranges or withdrawal of treatment), the patient did not require monitoring initially (i.e., patient deteriorated and required monitoring subsequently), the patient had less than 50% of the 24-hour epoch’s recording available, or the patient was monitored but the data was not recorded. The latter arises because patients can be often temporarily disconnected at different times of the day (e.g., washing and wheeling to imaging studies). In these cases, a member of the study team was required to manually reconnect and resume the recording.

### Statistical analysis

The analysis plan for this study was developed as part of a doctorate thesis investigating temporal profiles of brain physiological parameters following severe TBI and was determined beforehand, during the designing stages of the study. The analysis did not differ from the original plan; however, following the suggestion of reviewers, we performed one additional analysis (analysis plan attached as S2 Appendix). Descriptive statistics were used to summarise the study population. Data for continuous variables are presented as means with standard deviations (SDs) or medians with interquartile ranges (IQRs). Categorical data are presented as counts and frequencies. Continuous variables were compared between survivors and nonsurvivors using a *t* test or Wilcoxon Rank Sum test, depending on the normality of the data. Categorical data were compared using a Pearson’s chi-square test or Fisher's exact test. The raw means of ICP and PRx from T_24_ to T_240_ were plotted, and the difference between strata was calculated using an ANOVA test with Bonferroni post hoc adjustments.

Three complementary statistical approaches were employed to evaluate the impact of the temporal profile of ICP and PRx on fatal outcome. First, to assess the difference in brain physiological parameters and their trajectories between those with a fatal outcome and functional survivors, we utilized a linear mixed-effects model (LMEM) with a between-subjects factor (group: fatal versus nonfatal), a within-subject factor (time: T_24_ to T_240_), and the interaction between these 2 with patient ID as a random effect. An unstructured covariance structure provided the best model fit based on the Akaike Information Criterion. The following variables were used for adjusting the model: age, sex, best preintubation GCS, primary injury type (diffuse versus mass lesion), and surgical interventions (none, craniotomy, primary DC, and secondary DC). Since DC is a strong modulator of ICP and the “open skull model” may affect the reliability of the cerebrovascular reactivity assessment, an adaptive intervention parameter was used for the DC variable whereby the occurrence of the intervention over time was adjusted to the exact time point (T_24_ to T_240_) when the procedure took place. In addition to plotting the raw means of ICP and PRx over time, Bonferroni adjusted pairwise multiple comparisons of the estimated marginal means (EMMs) were generated with LMEMs. These means are calculated for the 2 outcome groups for each 24-hour epoch by taking into account all fixed and random effects variables used in the LMEM.

Second, a generalized linear mixed model (GLMM) was next used to examine the effect of ICP and PRx on the probability (odds ratio [OR]) of having a fatal outcome over time, using repeated measures of these parameters over the first 240 hours postinjury. The model also included the same fixed and random effects and interactions term as the LMEM model.

The third approach tested how well ICP and PRx could distinguish between fatal outcome and functional survivors during different time points (T_24_–T_240_); the area under the receiver operating characteristic curve (AUC-ROC) was calculated and compared. ROC curves are frequently used for displaying sensitivity and specificity of a continuous diagnostic marker (i.e., ICP or PRx) for a binary disease variable (fatal outcome yes/no). However, many disease markers and outcomes are time dependent, and ROC curves that vary as a function of time may be more appropriate. Therefore, we summarised the discrimination potential of ICP and PRx by calculating ROC curves for the cumulative effects of ICP and PRx by time (T_24_–T_240_) using GLMMs.

All statistical analyses were conducted using R statistical software version 3.2.3 [36] and Stata 14.2 SE (StataCorp, College Station, Texas, US). All statistical tests were performed with α ≤ 0.05 (2-tailed).

## Results

### Patient characteristics

A total of 601 severe TBI patients with high-resolution continuous monitoring were identified over the study period, with a cumulative sum of 92,737 hours of monitoring data following artefact clearing. The median (IQR) total time of monitoring for all patients was 126 (156) hours. Excluded 24-hour epochs (because of recordings covering < 50% of the 24-hour period) were further evaluated, and this evaluation demonstrated that most excluded epochs were in the first 24 hours, with a median recording duration of 4 hours (Table A–F in S2 Supporting Information). The mean (SD) age was 39 (17) years, 77% were males, and 70% of patients sustained diffuse brain injury (Table 1). The best preintubation GCS for the majority of patients was between 3 and 8. Forty percent of patients underwent a trauma craniotomy or craniectomy during their admission. Acute subdural haematomas (aSDHs) were the most frequently evacuated mass lesion in this cohort. Of those who underwent a DC, 50% of the patients underwent a primary DC for evacuation of a mass lesion, and the other 50% a secondary DC for refractory raised ICP. Almost all primary DCs were performed within the first day following injury, while on average secondary DCs were performed on the fourth day following injury (Table 1). A total of 113 patients (19%) had a fatal outcome within the study period, and the median (IQR) time from injury to death was 12 (13) days. The overall functional outcome according to the GOS is presented in Table 1. Almost one-fourth of the patients in this cohort with a fatal outcome died from non-neurological causes (i.e., respiratory failure, sepsis, or myocardial infarction). The baseline characteristics of those with a fatal outcome (due to nonsurvivable TBI or brain death) or functional survivors (severe disability, moderate disability, and good recovery) at 6 months postinjury were further stratified in Table 2. Patients with a fatal outcome due to neurological causes were older (45 ±18 years versus 38 ± 16 years, *p* < 0.001), had a lower preintubation GCS (81% versus 70% GCS of 3–8, *p* = 0.047), required more surgical interventions (56% versus 38%, *p* < 0.001), had a higher mean ICP (21.0 ± 10.2 mmHg versus 15.1 ± 8.2 mmHg, *p* < 0.001), a lower mean CPP (75.9 ± 8.3 mmHg versus 78.5 ± 8.0 mmHg, *p* = 0.009), a higher mean PRx (0.16 ± 0.21 a.u. versus 0.05 ± 0.15 a.u., *p* < 0.001), and spent more time in pathological ranges of ICP (>25 mmHg) (18.0% [32.7%] versus 2.60% [6.90%] of total monitoring time, *p* < 0.001) and PRx (>0.25) (42.9% [32.9%] versus 32.2% [19.6%] of total monitoring time, *p* < 0.001). No differences were observed in sex, primary injury type, or DC type. Also, the median monitoring time for fatal and nonfatal (*p* = 0.991) and the median time to primary and secondary DC (*p* = 0.893 and *p* = 0.558, respectively) did not differ between the 2 outcome groups.

**Table 1:** Baseline characteristics of 601 severe traumatic brain injury (sTBI) patients with high-resolution continuous monitoring, diagnosed between September 2002 and January 2016.

	*n*	%
**Mean age years (years ± SD)**	39 **±** 17	
**Sex**		
Female	137	23
Male	464	77
**Best preintubation GCS**		
3–8	435	72
9–15	166	28
**Primary injury type**		
Diffuse	423	70
Mass lesion	178	30
**Surgical interventions**		
No interventions	356	60
Craniotomy for mass lesion	73	12
Extradural	23	(32)
Acute subdural	38	(52)
ICH/contusion	12	(16)
Primary DC for mass lesion	86	14
Extradural	12	(14)
Acute subdural	63	(73)
ICH/contusion	11	(13)
Secondary DC for refractory ICP	86	14
**DC type**		
Bifrontal craniectomy	44	26
Hemicraniectomy	126	73
Posterior fossa decompression	2	1
**Mean time from injury to primary DC (median hours [IQR])**	6.0 [5.0]	
**Mean time from injury to secondary DC (median hours [IQR])**	45 [81]	
**Glasgow Outcome Scale at 6 months**		
Death	113	19
Vegetative state	20	3
Severe disability	203	34
Moderate disability	152	25
Good recovery	113	19
**Causes of death**		
Non-neurological cause	25	22
Nonsurvivable TBI or brain death	88	78
**Days from injury to death (median [IQR])**	12 [13]	
**Monitoring time in hours (median [IQR])**	126 [156]	

Abbreviations: DC, decompressive craniectomy; GCS, Glasgow Coma Scale; ICH, intracerebral hematoma; ICP, intracranial pressure; IQR, interquartile range; TBI, traumatic brain injury; SD, standard deviation. Values within parentheses represent subtable percentages.

**Table 2:** Baseline characteristics of 556 severe traumatic brain injury (sTBI) patients stratified by fatal outcome (due to nonsurvivable TBI or brain death) and functional survivors (ranging from severe disability to good recovery) at 6 months postinjury.

	Functional survivors (*n* = 468)	Fatal outcome (*n* = 88)	
	*N* (%)	*N* (%)	*p*-value†
**Age in years (mean ± SD)**	38 **±** 16	45 **±** 18	<0.001*
**Sex**			0.943
Female	108 (23)	20 (23)	
Male	360 (77)	68 (77)	
**Best preintubation GCS**			0.047*
3–8	329 (70)	71 (81)	
9–15	139 (30)	17 (19)	
**Primary injury type**			0.247
Diffuse	337 (72)	58 (66)	
Mass lesion	131 (28)	30 (34)	
**Surgical interventions**			<0.001*
No interventions	293 (62)	39 (44)	
Craniotomy for mass lesion	55 (12)	10 (12)	
Primary DC for mass lesion	56 (12)	23 (26)	
Secondary DC for refractory ICP	64 (14)	16 (18)	
**DC type**			0.465
Bifrontal craniectomy	34 (28)	8 (20)	
Hemicraniectomy	85 (71)	30 (77)	
Posterior fossa decompression	1 (1)	1 (3)	
**Hours from injury to primary DC (median [IQR])**	6.4 [6.4]	7.9 [6.3]	0.893
**Hours from injury to secondary DC (median [IQR])**	55 [96]	73 [137]	0.558
**Monitoring time in hours (median [IQR])**	124 [161]	146 [150]	0.991
**ICP mmHg (mean ± SD)**	15.1 ± 8.2	21.0 ± 10.2	<0.001*
**CPP mmHg (mean ± SD)**	78.5 ± 8.0	75.9 ± 8.3	0.009*
**PRx a.u. (mean ± SD)**	0.05 ± 0.15	0.16 ± 0.21	<0.001*
**% of monitoring ICP > 25 mmHg (median [IQR])**	2.60 [6.90]	18.0 [32.7]	<0.001*
**% of monitoring PRx > 0.25 a.u. (median [IQR])**	32.2 [19.6]	42.9 [32.9]	<0.001*

† *p*-values were calculated by *X*^2^-test for sex, GCS, injury, and DC type, and surgical intervention; by the Mann-Whitney U-test for time to DC, monitoring time, and % time > thresholds; and by a Student’s *t* test for age, ICP, CPP, and PRx.

*Statistically significant *p* < 0.05.

Abbreviations: 95% CI, 95% confidence interval; CPP, cerebral perfusion pressure; DC, decompressive craniectomy; GLMM, generalized linear mixed model; GCS, Glasgow Coma Scale; ICH, intracerebral hematoma; ICP, intracranial pressure; OR, odds ratio; PRx, pressure reactivity index; SD, standard deviation; TBI, traumatic brain injury.

### The course of ICP and PRx over time

**Fig 1:**
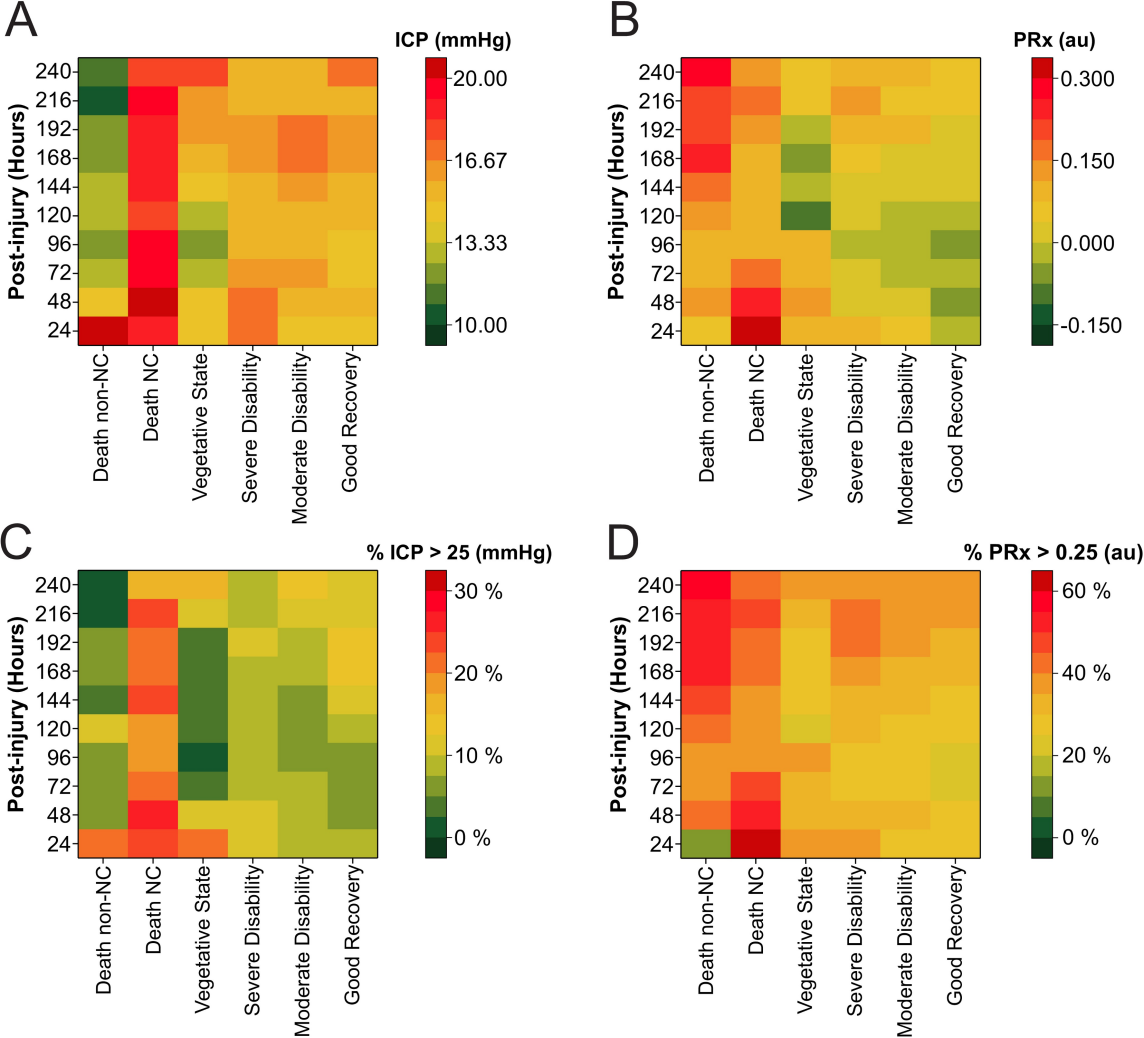
Heatmap illustrating levels of intracranial pressure (ICP) and pressure reactivity index (PRx) in 601 severe traumatic brain injury (TBI) patients stratified by different levels of functional outcome. The colour code represents (A) mean ICP, (B) mean PRx, (C) percent time spent ICP > 25 mmHg, and (D) percent time spent PRx > 0.25 for each 24-hour epoch following injury. Tabulated heatmaps are available as supporting information (Table A–D in S3 Supporting Information). Abbreviations: NC, neurological cause.

Heat maps showing the ICP and PRx parameters from 601 sTBI patients over the course of the first 240 hours postinjury are shown in Fig 1. The heat map showed that the high levels of ICP were associated with fatal outcome from neurological causes. In contrast, a fatal outcome due to non-neurological causes did not demonstrate raised ICP during the first 240 hours, except for the first 24-hour epoch. However, there were only 2 patients contributing data to this first time point. Levels of PRx in fatal outcome demonstrated that the 2 different causes of death showed differential patterns of cerebrovascular impairment. In the group experiencing fatal outcome from neurological causes, cerebrovascular pressure reactivity seemed to be impaired in the first 72 hours following the injury. However, patients with a fatal outcome from non-neurological causes demonstrated late impairment in pressure reactivity after 168 hours following injury, possibly due to the development of (multiple) organ dysfunction. No distinct patterns of ICP were observed to discriminate between different levels of functional outcome (severe disability–good recovery). The PRx heat map, however, did show a pattern of lower values of PRx with higher levels of functional outcome (severe disability compared to moderate disability or good recovery). Patients in a vegetative state did not demonstrate patterns different from other survivors; however, given the limited number of patients in this group and at each 24-hour time point, the interpretation of these patterns has to be done cautiously. Physiologically “abnormal” thresholds of ICP (>25 mm Hg) and PRx (>0.25 a.u.) were visualised in Fig 1C and 1D and were based on the recent RESCUEicp trial, which used a threshold of 25 mm Hg for intracranial hypertension, and studies that found a PRx of >0.25 gave the best statistical separation between outcomes [14,19].

### Observed and estimated means by outcome

**Fig 2:**
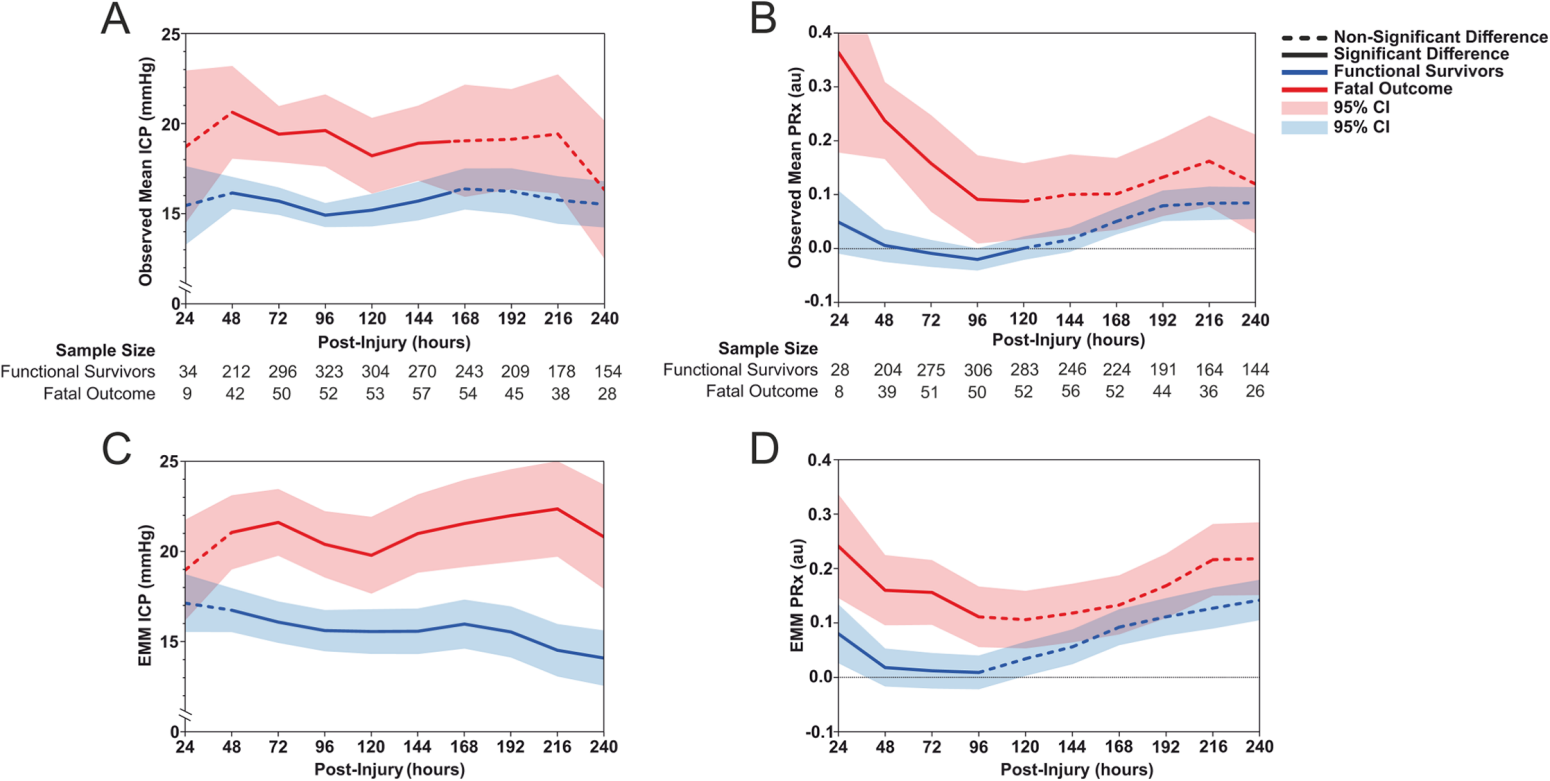
**Observed mean values of (A) intracranial pressure (ICP) and (B) pressure reactivity index (PRx) of 556 traumatic brain injury (TBI) patients stratified by functional survivors and fatal outcome due to neurological causes for each 24-hour epoch for the first 240 hours after injury. The estimated marginal means (EMMs) of (C) ICP and (D) PRx are plotted over the same strata as derived from the repeated-measures generalized linear mixed model (GLMM) after adjusting for patient, injury, and treatment characteristics**.

As expected, in the immediate postinjury period the number of patients in the first 24 hours postinjury is limited (Fig 2A and 2B). In terms of patients experiencing a fatal outcome, it is unlikely that a disproportionate number of patients expiring in the early time points (Fig 2A and 2B) is giving a false impression of time course: the number of patients with a fatal outcome contributing to each 24-hour epoch remained relatively stable over the first 10 days following injury. There is no peak incidence of death at any time point, and the number of deaths remains proportional to the nonfatal group (Fig 2A and 2B). Mean ICP was on average higher than 20 mmHg 48 hours after injury only in those with a fatal outcome (Fig 2A). The difference in mean (95% CI) ICP between those with a fatal outcome and functional survivors was only significant for the first 168 hours after injury (Fig 2A: T_48_ 16.1 [15.3–17.0] versus 20.6 [18.1–23.2], T_72_ 15.7 [14.9–16.5] versus 19.4 [17.9–21.0], T_96_ 14.9 [14.2–15.6] versus 19.6 [17.6–21.6], T_120_ 15.2 [14.3–16.1] versus 18.2 [16.1–20.3], T_144_ 15.7 [14.6–16.8] versus 18.9 [16.8–21.0], T_168_ 16.4 [15.2–17.5] versus 19.0 [15.9–22.2], all *p* < 0.05). While the time course of the mean ICP was relatively flat, 2 minor peaks in ICP can be appreciated from 48–72 hours and at 216 hours (Fig 2A and 2C). Mean (95% CI) values of PRx demonstrated significantly higher (impaired) levels in those with a fatal outcome during the first 120 hours postinjury (Fig 2B: T_24_ 0.05 [−0.01 to 0.11] versus 0.36 [0.18–0.55], T_48_ 0.01 [−0.02 to 0.04] versus 0.24 [0.17–0.31], T_72_ −0.01 [−0.03 to 0.02] versus 0.16 [0.07–0.25], T_96_ −0.02 [−0.04 to 0.00] versus 0.09 [0.01–0.17], T_120_ 0.00 [−0.02 to 0.02] versus 0.09 [0.02–0.16], all *p* < 0.05), with abnormally high values observed mainly in the first 48 hours postinjury.

The estimated means from the LMEM showed the difference in the trajectory of fatal outcome patients and functional survivors across the first 240 hours postinjury after adjusting for patient, injury, and treatment characteristics. The estimated means of ICP demonstrated that ICP levels were significantly different at all time points between the 2 outcome groups, except for the first 24 hours (Fig 2C). Patients with a fatal outcome had ICP values above 20 mmHg for all time points after the first 24 hours. Functional survivors showed low levels of ICP throughout the first 240 hours, with a relatively steady decrease over time. The estimated means of PRx demonstrated only significant differences between the patients with fatal outcomes and those with nonfatal outcomes during the first 96 hours following injury (Fig 2D). While the trajectories of the estimated means of PRx are relatively similar to the observed means, a smaller separation of PRx values between the fatal and nonfatal groups can be noted.

### Generalized linear mixed model

In addition to the dynamic variables (ICP, PRx, CPP, and surgical interventions), patients’ baseline and injury characteristics were considered as potential prognostic variables and were included in the GLMM (Table 3). The results of this analysis identified age at injury (OR 1.05 [95% CI 1.02–1.07, *p* < 0.001]); best preintubation GCS: a higher GCS score demonstrated a lower likelihood of a fatal outcome (OR 0.60 [95% CI 0.45–0.80, *p* < 0.001]); and primary injury type with mass lesions demonstrating a lower likelihood of a fatal outcome when compared to diffuse injuries (OR 0.20 [95% CI 0.12–0.34, *p* < 0.001]); the results also revealed that patients requiring surgical interventions demonstrated a higher likelihood of fatal outcome, with craniotomy for mass lesions (OR 6.43 [95% CI 3.56–11.60]), primary DC for mass lesion (OR 13.19 [95% CI 7.60–22.89]), and secondary DC for refractory ICP (OR 1.48 [95% CI 1.05–2.07]) (all *p* ≤ 0.05), ICP (OR 1.19 [95% CI 1.12–1.25, *p* < 0.001]), and PRx (OR 11.43 [95% CI 2.84–45.92, *p* = 0.001]) shown as being associated with fatal outcome (Table 3). Sex and CPP were not significantly associated with outcome.

**Table 3:** A generalized linear mixed model (GLMM) was used to test for associations between the independent variables and fatal outcome by taking into account repeated measures and their interactions with time.

	GLMM analysis	
	OR (95% CI)	*p*-value†
**Age in years**	1.05 (1.02–1.07)	<0.001*
**Sex**		0.422
Female	Ref	
Male	0.89 (0.68–1.18)	
**Best preintubation GCS**		<0.001*
3–8	Ref	
9–15	0.60 (0.45–0.80)	
**Primary injury type**		<0.001*
Diffuse	Ref	
Mass lesion	0.20 (0.12–0.34)	
**Surgical interventions**		0.006*
No interventions	REF	
Craniotomy for mass lesion	6.43 (3.56–11.60)	
Primary DC for mass lesion	13.19 (7.60–22.89)	
Secondary DC for refractory ICP	1.48 (1.05–2.07)	
**ICP mmHg**	1.19 (1.12–1.25)	<0.001*
**CPP mmHg**	0.98 (0.93–1.05)	0.594
**PRx a.u.**	11.43 (2.84–45.92)	0.001*

† *p*-values were calculated using a GLMM.

*Statistically significant *p* < 0.05.

Abbreviations: 95% CI, 95% confidence interval; CPP, cerebral perfusion pressure; DC, decompressive craniectomy; GCS, Glasgow Coma Scale; GLMM, generalized linear mixed model; ICH, intracerebral hematoma; ICP, intracranial pressure; OR, odds ratio; PRx, pressure reactivity index; Ref, reference group; SD, standard deviation; TBI, traumatic brain injury.

### Evolution of AUC-ROC

**Fig 3:**
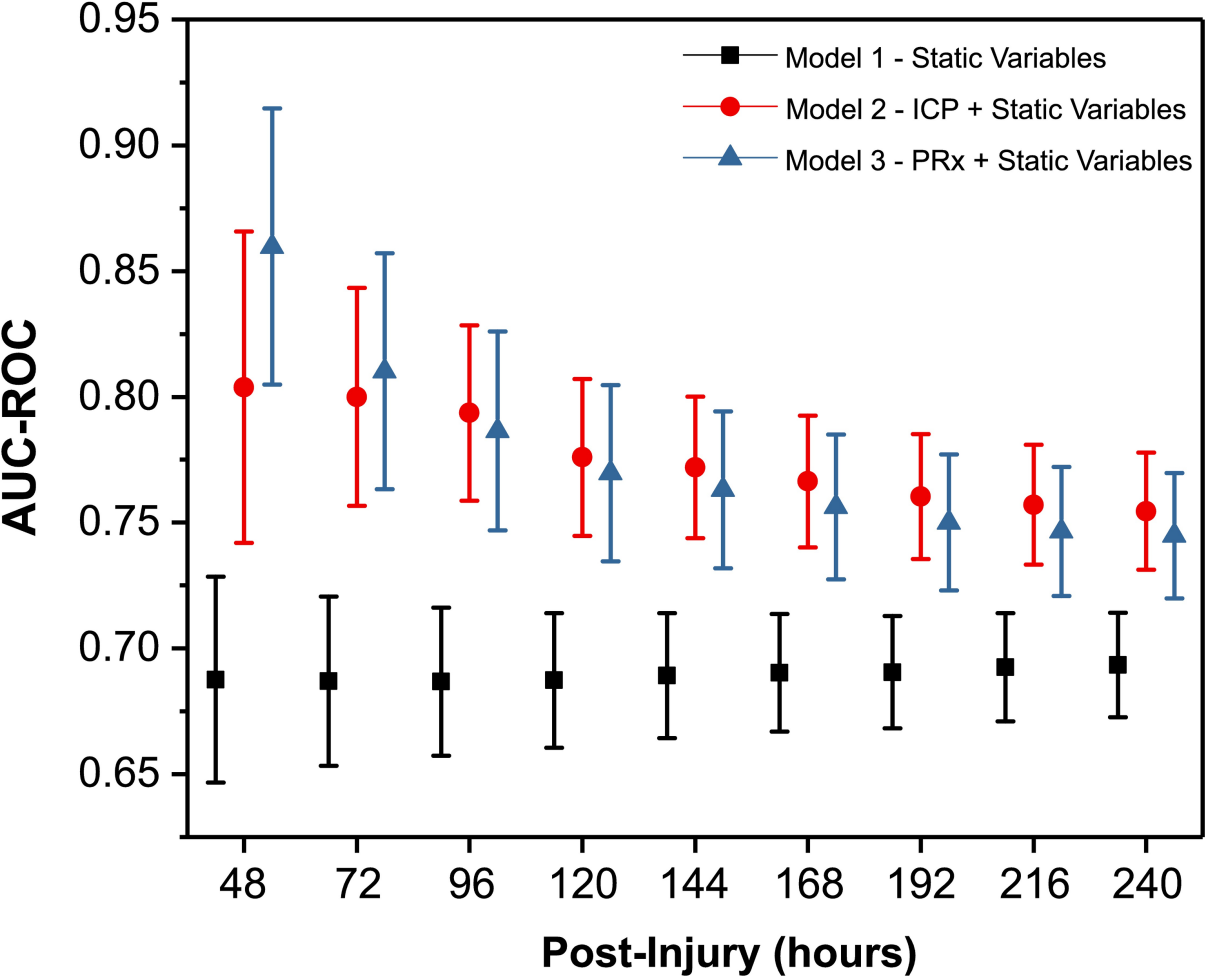
Time-dependent receiver operating characteristic (ROC) curve analysis for prediction of fatal outcome due to neurological causes. The evolution of the area under the ROC curve (AUC-ROC) of 3 different models over time has been plotted. Generalized linear mixed model (GLMM) Model 1: static (constant) variables with patient, injury, and treatment characteristics. GLMM Model 2: repeated measures of intracranial pressure (ICP) including static variables from Model 1. GLMM Model 3: repeated measures of pressure reactivity index (PRx) including static variables from Model 1. At each time point, GLMM Models 2 and 3 are using repeated measures of the dynamic variables (ICP and PRx) from baseline to the respective time point. Due to the limited sample size for T_24_, data have not calculated and plotted for this time point separately. The tabulated graph is available as supporting information (Table A in S4 Supporting Information).

The predictive ability of 3 different models to distinguish death from neurological causes versus survival is shown in Fig 3. Model 1 uses a GLMM to predict fatal outcome using only the traditional static (or constant) clinical predictive variables such as age, sex, preintubation GCS, primary injury type, and surgical interventions. This model was relatively constant (with an AUC-ROC of approximately 0.69) throughout the first 10 days postinjury owing to the fact that most of these variables remain constant over time, and only small variations will be contributed by the addition of patients as time increases and more patients are added to the model. Model 2 utilised the same GLMM from Model 1 but combined the constant prognostic factors with the cumulative addition of the dynamic monitoring variable, ICP. The addition of ICP to the model significantly improved the ability to distinguish death due to neurological causes from survival. Although the AUC-ROC for the ICP model was highest from day 0 to day 2 (AUC-ROC = 0.80; 95% CI 0.74–0.87), the AUC-ROC was not significantly lower with the accumulation of ICP data from 0–240 hours, as indicated by overlapping AUC-ROC values and their confidence intervals (all *p* > 0.05). The third model incorporated the constant prognostic factors (GLMM Model 1) with the cumulative addition of the dynamic monitoring variable PRx and demonstrated a superior predictive ability (AUC-ROC = 0.86; 95% CI 0.81–0.92) using data from the first 48 hours postinjury when compared to the first 120 to 240 hours (AUC-ROC = 0.77; 95% CI 0.73–0.80, to AUC-ROC = 0.74; 95% CI 0.72–0.77 [all *p* < 0.05]).

## Discussion

In a cohort study of 601 patients with sTBI, we demonstrate that despite relatively stable ICP throughout the first 10 days postinjury, cerebral pressure reactivity is impaired early after injury. Furthermore, studying the prognostic importance of brain physiological parameters after separating neurological from non-neurological causes of death resulted in a strong relationship of both ICP and pressure reactivity with fatal outcome from neurological causes, which is clinically meaningful and arguably more relevant. Finally, the inclusion of ICP and pressure reactivity into a dynamic predictive model demonstrated the importance of the temporal profile of these parameters; inclusion of ICP or pressure reactivity significantly improved our ability to predict patient outcome when compared to static variables. Currently, the most commonly used TBI prediction models only utilise fixed variables [37,38].

### ICP and PRx evolution

While many studies document ICP monitoring data after TBI, the vast majority of these neglect the time-varying nature of the signal. Published studies seem to agree that ICP increases at some point after injury, but the timing remains elusive, with 1–3, 3–5 and 7–14 days all being proposed as time frames [9,10,13,14,20]. The causes of this increased ICP after TBI could be evolving cerebral oedema, changes in cerebral blood volume, or the development of mass lesions [39–41].

In the current study, we found an increased ICP in those with death from neurological causes in both the raw ICP and adjusted ICP profile (Figs 1 and 2), supporting the concept that ICP is an important contributor to harmful secondary brain injuries. Of note, the increased ICP in the nonsurvivors was apparent in most cases well before the patient death (ICP increased on day 1, and the average time of patient death is 17 days) (Table 1). However, in the current study we did not find a strong temporal evolution of ICP, despite the large sample size and the accurate determination of the time of injury (Figs 1 and 2). Heterogeneity of TBI pathophysiology could be contributing to this absence of a clear temporal profile. Furthermore, as clinical protocols have developed in which control of ICP is the central purpose of sTBI management, it is likely that some of the information that is reflected in the natural history of ICP changes is no longer apparent because of therapeutic intervention. As such, a raised ICP in this patient cohort represents a failure to control ICP with the medical and surgical means at the treating physician’s disposal. This lack of a clear time course is consistent with the disparate timings of increased ICP reported in previous, smaller investigations and underscores the fact that predicting the timing of increased ICP based purely on time of monitoring is problematic; increased ICP can occur at any time in the acute management phase and is uniformly deleterious.

Cerebral pressure reactivity was both impaired early (0–3 days) and showed a deteriorating course later (5–10 days) after injury (Fig 2B and 2D); however, this first impairment was only observed in the patients who subsequently died because of neurological causes. This early pressure reactivity derangement possibly implicates vascular impairment stemming from the primary injury and could have relevant therapeutic and research implications; a therapy aiming to optimise cerebrovascular function after TBI should focus on the first 3 days after injury, as should novel biomarkers or noninvasive estimates of cerebrovascular impairment.

As the second (delayed) pattern of worsening pressure reactivity is present in both the survival and mortality outcome groups, it appears to have less clinical relevance and may not necessarily be a sensitive marker of cerebral injury. Explanations of this temporal evolution are, however, difficult; in contrast to the scant studies on the time course of ICP after injury, this is the only study (to our knowledge) to assess continuous cerebral vascular reactivity in relation to time from injury. In contrast to our findings, investigations intermittently assessing pressure autoregulation using transcranial Doppler have found impaired pressure autoregulation in the first 4–5 days postinjury before a gradual return towards normal values [42–45]. Cerebrovascular reactivity can be seen as a proxy variable of various neuropathophysiological processes and interventions that can modulate it. Secondary worsening in cerebral pressure reactivity could also be influenced by treatments administered during the monitoring period, including alterations in PaCO_2_ during ventilator weaning [43,46], changes in pharmacological sedation [47], or temperature management [48]. Secondary worsening could also be a sequelae pathophysiological processes, i.e., the development of post-traumatic vasospasm, which has been shown to occur in approximately 40% of TBI patients and presents with a not dissimilar time course [49]. Fig 3 demonstrated statistically better predictive ability of PRx in the first 48 hours postinjury when compared to the first 120 to 240 hours. Therefore, this finding may suggest that PRx impairment in the first 2 days is reflecting pathophysiological processes with good discriminatory power for fatal outcome; however, from day 5 onwards, the pathophysiological pathways that modulate PRx might not be the same ones from the first days and therefore might not be able to discriminate fatal outcome with the same power, leading to a decreasing evolution of the AUC-ROC over time.

### Neurological monitoring is related to neurological outcome

In the current study, patients who died because of neurological causes showed distinctly different ICP and pressure reactivity time courses when compared to those who died because of non-neurological causes (Fig 1). While traditionally studies relate monitored variables to all-cause mortality or GOS, using such nonspecific outcome measures may obscure important relationships. Acknowledging that the current study did not use a validated instrument to assess neuronal specific injury, the relatively crude method of dichotomizing patients who died into neurologic and non-neurologic causes nevertheless provided fruitful; almost a quarter of the patients who died did so because of non-neurologic causes, and in these patients, both ICP and pressure reactivity over the first 5 days were normal. This distinction may be especially relevant in cases of multitrauma.

Interestingly, pressure reactivity 5–10 days after injury is markedly increased in those who died due to non-neurological causes. This perhaps reflects the multivariate nature of vascular dysregulation; indeed, previous investigations have shown disturbed cerebral vascular function during seemingly non-neurological scenarios such as impaired arterial glucose regulation, impaired kidney function, or after red blood cell transfusion [50–52]. In addition, common conditions in this group included sepsis, respiratory failure, and postresuscitation from cardiac arrest, all of which have been shown to be characterised by impaired cerebrovascular reactivity and resulting in high short-term mortality [53–56].

### ICP and PRx monitoring as a time-driven adaptive outcome prediction tool

ICP and PRx monitoring can facilitate reasonably accurate, personalised, and dynamic assessments of patient prognosis. In our analysis, the explanatory power of the PRx decreases over time; therefore, the prognostic weight assigned to PRx should similarly decrease (Fig 3). For ICP, no significant changes were seen over time for its ability to predict fatal outcome. By accumulating information regarding secondary injuries from intracranial hypertension or cerebrovascular dysregulation, an adaptive model has the potential to inform therapy intensity, assess treatment efficacy, and provide up-to-date prognostic information for clinical use.

Furthermore, because ICP and ABP are the cornerstones of monitoring after sTBI, the inclusion of ICP and PRx into an adaptive model in most cases requires no additional inconvenience or harm to the patient. The inclusion of the monitoring data merely makes effective use of data that might otherwise be discarded. While the current study focussed on ICP and PRx monitoring data, it is conceivable that the addition of other neuromonitoring data that address distinct pathophysiological processes may further improve the accuracy of the model. In this regard, dynamic markers of intracranial compliance, autonomic nervous system activity, or cerebral metabolism may be useful adjuncts [7,57,58]

Rather than aiming to replace existing prognostic models, such as the Corticosteroid Randomisation after Significant Head Injury (CRASH) and International Mission for Prognosis and Analysis of Clinical Trials in TBI (IMPACT-TBI) models, which have been shown to have good discriminating ability [37,38], the purpose of this predictive model was to assess the value of including knowledge about the patient's state of current physiology. Such an adaptive model may more closely align with clinical acumen and Bayesian inferencing, whereby estimation of outcome is informed by the prior information (from the constant predictive variables) and updated with new information from the monitored predictors.

### Limitations

The current study has certain limitations. First, in the current cohort, measurement of ICP and PRx over all 10 days was not possible for all patients: some patients required a short period of monitoring (i.e., because of ICP within normal ranges), some did not require monitoring initially (i.e., patient deteriorated and required monitoring later on), and in some, disconnections from the recording hardware occurred. This, however, reflects the clinical reality of neuromonitoring after sTBI. Also, measurements of pupil reactivity were not reliably obtainable for all patients in this cohort, as prognostic models for sTBI derived from the CRASH and IMPACT studies both support the use of pupil reactivity in building prognostic models for patient outcome based on initial patient characteristics [37,38]. Since the study period spanned over 13 years, changes in treatment protocols over the study period had to be tested by adding a term for calendar time in our models, demonstrating no significant effect on our outcome measures. The inevitable updating of the protocol is unlikely to influence the primary findings of this study—i.e., that temporal profiles of ICP and PRx differ according to fatal outcome. This is because across the entire study period, intracranial hypertension was vigorously treated and did not vary depending on how many days the patient was postinjury.

The observed time courses of ICP and PRx could be due to the interventions received during their NCCU stay and not the natural history of the disease. These parameters are modulated by various treatments and in the case of ICP directly targeted (as part of tiered protocols) to be maintained within certain limits. As PRx is not directly targeted using our current therapeutic strategies, this may explain why it provides additional prognostic information compared to the target-driven control of ICP. We have adjusted our analyses for the injury type and surgical treatments and particularly focused on DC by using a time-driven DC variable. Since DC has been shown to lead to a dramatic reduction in ICP and influence CR, we have adjusted all repeated measures of ICP and PRx in our LMEM and GLMM for the occurrence of DC [59]. It would have been advantageous to adjust for the intensity of other intervention parameters (hyperosmolar therapies, hyperventilation, and hypothermia); however, these data were not available for our cohort.

Although this is the largest evaluation of the temporal profile of neuromonitoring variables to date, it has been conducted at a single institution. Thus, the current findings describing temporal evolution of neuromonitoring data and the adaptive prognostic model should be externally validated in a multicentre prospective study.

Finally, while the evolution of ICP and pressure reactivity has been described, the underlying pathophysiology of these profiles remains occult. Building on this temporal approach, further work should investigate treatment effects and their relationship with other aspects of patient pathology and physiology. Both parameters carry potential to provide utility as predictive parameters because they represent the dysregulation of pathways directly involved in pathogenesis [60,61]. However, further studies are needed not only to characterise these parameters as descriptive biomarkers (reflecting only the state/severity of the injury) and/or as actionable biomarkers (to guide clinical management and measure treatment response) but also to study how their temporal profile affects these characteristics.

### Conclusions

In this large single-centre cohort study, we demonstrate the temporal evolution of ICP and cerebral pressure reactivity index, indicating a potential early prognostic and therapeutic window. By distinguishing neurological from non-neurological causes of death, robust relationships of ICP and PRx and their time course with outcome were obtained. Finally, the combination of static clinical prognostic factors and dynamic monitoring variables contributed to a significantly better prediction of outcome. Therefore, time-driven dynamic modelling of outcome in sTBI patients may allow for more accurate, temporally relevant, and clinically useful prediction models.

## Supporting information

S1 Appendix Transparent reporting of a multivariable prediction model for individual prognosis or diagnosis (TRIPOD) checklist. (PDF)

S2 Appendix Original analysis plan and modifications following comments from editors and reviewers. (PDF)

S1 Supporting Information Traumatic brain injury ICP/CPP algorithm at the neurosciences and trauma critical care unit, Addenbrooke’s Hospital, Cambridge, UK. (PDF)

S2 Supporting Information Patient data excluded due to low coverage of 24-hour epoch. (PDF)

S3 Supporting Information Tabulated heatmaps from Fig 1A–1D. (PDF)

S4 Supporting Information Tabulated data from Fig 3 (time-dependent AUC-ROC). (PDF)

## Abbreviations

ABP: arterial blood pressure
AUC: area under the ROC curve
aSDH: acute subdural haematoma
CBF: cerebral blood flow
CPP: cerebral perfusion pressure
CRASH: Corticosteroid Randomisation after Significant Head Injury
DC: decompressive craniectomy
EMM: estimated marginal mean
GCS: Glasgow Coma Scale
GLMM: generalized linear mixed model
GOS: Glasgow Outcome Scale
ICH: intracerebral hematoma
ICP: intracranial pressure
IMPACT-TBI: International Mission for Prognosis and Analysis of Clinical Trials in TBI
IQR: interquartile range
LMEM: linear mixed-effects model
NCCU: Neuro Critical Care Unit
OR: odds ratio
PRx: pressure reactivity index
RESCUEicp: Randomized Evaluation of Surgery with Craniectomy for Uncontrollable Elevation of Intracranial Pressure
ROC: receiver operating characteristic
SD: standard deviation
sTBI: severe traumatic brain injury
TBI: traumatic brain injury
TRIPOD: Transparent Reporting of a multivariable prediction model for Individual Prognosis Or Diagnosis

## References

[1] Roozenbeek B, Maas AIR, Menon DK. Changing patterns in the epidemiology of traumatic brain injury. Nat Rev Neurol. 2013;9: 231–6. doi: 10.1038/nrneurol.2013.22 23443846 10.1038/nrneurol.2013.22

[2] Tagliaferri F, Compagnone C, Korsic M, Servadei F, Kraus J. A systematic review of brain injury epidemiology in Europe. Acta Neurochir (Wien). 2006;148: 255–68; discussion 268. doi: 10.1007/s00701-005-0651-y 16311842 10.1007/s00701-005-0651-y

[3] Stein SC, Georgoff P, Meghan S, Mizra K, Sonnad SS. 150 Years of Treating Severe Traumatic Brain Injury: a Systematic Review of Progress in Mortality. J Neurotrauma. 2010;27: 1343–53. doi: 10.1089/neu.2009.1206 20392140 10.1089/neu.2009.1206

[4] Maas AIR, Menon DK. Traumatic brain injury: Rethinking ideas and approaches. Lancet Neurol. 2012;11: 12–13. doi: 10.1016/S1474-4422(11)70267-8 22172614 10.1016/S1474-4422(11)70267-8

[5] Czosnyka M, Balestreri M, Steiner L, Smielewski P, Hutchinson PJ, Matta B, et al Age, intracranial pressure, autoregulation, and outcome after brain trauma. J Neurosurg. 2005;102: 450–4. doi: 10.3171/jns.2005.102.3.0450 15796378 10.3171/jns.2005.102.3.0450

[6] Güiza F, Depreitere B, Piper I, Citerio G, Chambers I, Jones PA, et al Visualizing the pressure and time burden of intracranial hypertension in adult and paediatric traumatic brain injury. Intensive Care Med. 2015;41: 1067–1076. doi: 10.1007/s00134-015-3806-1 25894624 10.1007/s00134-015-3806-1

[7] Timofeev I, Carpenter KLH, Nortje J, Al-Rawi PG, O’Connell MT, Czosnyka M, et al Cerebral extracellular chemistry and outcome following traumatic brain injury: a microdialysis study of 223 patients. Brain. Oxford University Press; 2011;134: 484–494. doi: 10.1093/brain/awq353 21247930 10.1093/brain/awq353

[8] Czosnyka M, Smielewski P, Kirkpatrick P, Menon DK, Pickard JD. Monitoring of cerebral autoregulation in head-injured patients. Stroke. 1996;27: 1829–1834. doi: 10.1161/01.STR.27.10.1829 8841340 10.1161/01.str.27.10.1829

[9] Menon DK. Cerebral protection in severe brain injury: physiological determinants of outcome and their optimisation. Br Med Bull. 1999;55: 226–58. 10695089 10.1258/0007142991902231

[10] Lundberg N. Continuous recording and control of ventricular fluid pressure in neurosurgical practice. Acta Psychiatr Scand Suppl. 1959;36: 1–193. 13764297

[11] Guillaume J, Janny P. Continuous intracranial manometry; physiopathologic and clinical significance of the method. Presse Med. 1951;59: 953 14864407

[12] Chesnut RM, Temkin N, Carney N, Dikmen S, Rondina C, Videtta W, et al A Trial of Intracranial-Pressure Monitoring in Traumatic Brain Injury. N Engl J Med. 2012;367: 2471–2481. doi: 10.1056/NEJMoa1207363 23234472 10.1056/NEJMoa1207363 PMC3565432

[13] Hutchinson P, Kolias A, Czosnyka M, Kirkpatrick P, Pickard J, Menon D. Intracranial pressure monitoring in severe traumatic brain injury. Bmj. 2013;346: f1000 doi: 10.1136/bmj.f1000 23418278 10.1136/bmj.f1000

[14] Hutchinson PJ, Kolias AG, Timofeev IS, Corteen EA, Czosnyka M, Timothy J, et al Trial of Decompressive Craniectomy for Traumatic Intracranial Hypertension. N Engl J Med. Massachusetts Medical Society; 2016;375: 1119–1130. doi: 10.1056/NEJMoa1605215 27602507 10.1056/NEJMoa1605215

[15] Overgaard J, Tweed W. Cerebral circulation after head injury. 1. Cerebral blood flow and its regulation after closed head injury with emphasis on clinical correlations. J Neurosurg. 1974;41: 531–541. doi: 10.3171/jns.1974.41.5.0531 4418221 10.3171/jns.1974.41.5.0531

[16] Fieschi C, Battistini N, Beduschi a, Boselli L, Rossanda M. Regional cerebral blood flow and intraventricular pressure in acute head injuries. J Neurol Neurosurg Psychiatry. 1974;37: 1378–1388. 4375173 10.1136/jnnp.37.12.1378 PMC1083656

[17] Czosnyka M, Smielewski P, Kirkpatrick P, Laing RJ, Menon D, Pickard JD. Continuous assessment of the cerebral vasomotor reactivity in head injury. Neurosurgery. 1997;41: 11-17-19. 10.1097/00006123-199707000-00005 9218290

[18] Donnelly J, Budohoski KP, Smielewski P, Czosnyka M. Regulation of the cerebral circulation: bedside assessment and clinical implications. Crit Care. 2016;20: 129 doi: 10.1186/s13054-016-1293-6 27145751 10.1186/s13054-016-1293-6 PMC4857376

[19] Sorrentino E, Diedler J, Kasprowicz M, Budohoski KP, Haubrich C, Smielewski P, et al Critical thresholds for cerebrovascular reactivity after traumatic brain injury. Neurocrit Care. 2012;16: 258–66. doi: 10.1007/s12028-011-9630-8 21964774 10.1007/s12028-011-9630-8

[20] Johnson U, Lewén A, Ronne-Engström E, Howells T, Enblad P. Should the Neurointensive Care Management of Traumatic Brain Injury Patients be Individualized According to Autoregulation Status and Injury Subtype? Neurocrit Care. 2014; doi: 10.1007/s12028-014-9954-2 24515639 10.1007/s12028-014-9954-2

[21] Dias C, Silva MJ, Pereira E, Monteiro E, Maia I, Barbosa S, et al Optimal Cerebral Perfusion Pressure Management at Bedside: A Single-Center Pilot Study. Neurocrit Care. 2015; doi: 10.1007/s12028-014-0103-8 25566826 10.1007/s12028-014-0103-8

[22] Steiner LA, Czosnyka M, Piechnik SK, Smielewski P, Chatfield D, Menon DK, et al Continuous monitoring of cerebrovascular pressure reactivity allows determination of optimal cerebral perfusion pressure in patients with traumatic brain injury. Crit Care Med. 2002;30: 733–738. doi: 10.1097/00003246-200204000-00002 11940737 10.1097/00003246-200204000-00002

[23] Aries MJH, Czosnyka M, Budohoski KP, Steiner L a., Lavinio A, Kolias AG, et al Continuous determination of optimal cerebral perfusion pressure in traumatic brain injury*. Crit Care Med. 2012;40: 2456–2463. doi: 10.1097/CCM.0b013e3182514eb6 22622398 10.1097/CCM.0b013e3182514eb6

[24] Stocchetti N, Colombo A, Ortolano F, Videtta W, Marchesi R, Longhi L, et al Time course of intracranial hypertension after traumatic brain injury. J Neurotrauma. 2007;24: 1339–1346. doi: 10.1089/neu.2007.0300 17711395 10.1089/neu.2007.0300

[25] Stein DM, Brenner M, Hu PF, Yang S, Hall EC, Stansbury LG, et al Timing of intracranial hypertension following severe traumatic brain injury. Neurocrit Care. 2013;18: 332–40. doi: 10.1007/s12028-013-9832-3 23494545 10.1007/s12028-013-9832-3

[26] Martin NA, Patwardhan R V, Alexander MJ, Africk CZ, Lee JH, Shalmon E, et al Characterization of cerebral hemodynamic phases following severe head trauma: hypoperfusion, hyperemia, and vasospasm. J Neurosurg. 1997;87: 9–19. doi: 10.3171/jns.1997.87.1.0009 9202259 10.3171/jns.1997.87.1.0009

[27] Balestreri M, Czosnyka M, Steiner L a, Schmidt E, Smielewski P, Matta B, et al Intracranial hypertension: what additional information can be derived from ICP waveform after head injury? Acta Neurochir (Wien). 2004;146: 131–41. doi: 10.1007/s00701-003-0187-y 14963745 10.1007/s00701-003-0187-y

[28] Souter MJ, Andrews PJ, Pereirinha MR, Signorini DF, Jones PA, Miller JD. Delayed intracranial hypertension: relationship to leukocyte count. Crit Care Med. 1999;27: 177–181. 9934913 10.1097/00003246-199901000-00048

[29] Robertson CS. Management of cerebral perfusion pressure after traumatic brain injury. Anesthesiology. 2001;95: 1513–1517. doi: 10.1055/s-2001-13835 11748413 10.1097/00000542-200112000-00034

[30] Coles JP, Minhas PS, Fryer TD, Smielewski P, Aigbirihio F, Donovan T, et al Effect of hyperventilation on cerebral blood flow in traumatic head injury: Clinical relevance and monitoring correlates*. Crit Care Med. 2002;30: 1950–1959. doi: 10.1097/01.CCM.0000026331.91456.9A 12352026 10.1097/00003246-200209000-00002

[31] Moons KGM, Altman DG, Reitsma JB, Collins GS. New guideline for the reporting of studies developing, validating, or updating a multivariable clinical prediction model: the TRIPOD statement. Adv Anat Pathol. 2015; doi: 10.1097/PAP.0000000000000072 26262512 10.1097/PAP.0000000000000072

[32] Katzenberger RJ, Ganetzky B, Wassarman DA. The gut reaction to traumatic brain injury. Fly (Austin). Taylor & Francis; 2015;9: 68–74. doi: 10.1080/19336934.2015.1085623 26291482 10.1080/19336934.2015.1085623 PMC5019014

[33] Pfeifer R, Teuben M, Andruszkow H, Barkatali BM, Pape H-C, Stefan P. Mortality Patterns in Patients with Multiple Trauma: A Systematic Review of Autopsy Studies. van Griensven M, editor. PLoS ONE. 2016;11: e0148844 doi: 10.1371/journal.pone.0148844 26871937 10.1371/journal.pone.0148844 PMC4752312

[34] Salehpour F, Bazzazi AM, Porhomayon J, Nader ND. Correlation between coagulopathy and outcome in severe head trauma in neurointensive care and trauma units. J Crit Care. 2011;26: 352–356. doi: 10.1016/j.jcrc.2010.12.005 21273031 10.1016/j.jcrc.2010.12.005

[35] Jennett B, Bond M. Assessment of outcome after severe brain damage: a practical scale. Lancet. 1975;II: 480–484. 10.1016/s0140-6736(75)92830-5 46957

[36] R Development Core Team. R: A language and environment for statistical computing. R Foundation for Statistical Computing, Vienna, Austria. URL http://www.R-project.org/. R Foundation for Statistical Computing, Vienna, Austria. 2013.

[37] Steyerberg EW, Mushkudiani N, Perel P, Butcher I, Lu J, McHugh GS, et al Predicting outcome after traumatic brain injury: development and international validation of prognostic scores based on admission characteristics. PLoS Med. 2008;5: e165; discussion e165. doi: 10.1371/journal.pmed.0050165 18684008 10.1371/journal.pmed.0050165 PMC2494563

[38] Perel P, Arango M, Clayton T, Edwards P, Komolafe E, Poccock S, et al Predicting outcome after traumatic brain injury: practical prognostic models based on large cohort of international patients. BMJ. 2008;336: 425–429. doi: 10.1136/bmj.39461.643438.25 18270239 10.1136/bmj.39461.643438.25 PMC2249681

[39] Stocchetti N, Maas AIR. Traumatic intracranial hypertension. N Engl J Med. 2014;370: 2121–30. doi: 10.1056/NEJMra1208708 24869722 10.1056/NEJMra1208708

[40] Choi S, Ph D, Brooks D, Lutz HA, Moulton RJ, Muizelaar JP, et al Contribution of CSF and vascular factors to elevation of ICP in severely head-injured patients. J Neurosurg. 1987;66: 883–890. doi: 10.3171/jns.1987.66.6.0883 3572518 10.3171/jns.1987.66.6.0883

[41] Maas AI, Stocchetti N, Bullock R. Moderate and severe traumatic brain injury in adults. Lancet Neurol. 2008;7: 728–741. doi: 10.1016/S1474-4422(08)70164-9 18635021 10.1016/S1474-4422(08)70164-9

[42] Hlatky R, Furuya Y, Valadka AB, Gonzalez J, Chacko A, Mizutani Y, et al Dynamic autoregulatory response after severe head injury. J Neurosurg. Journal of Neurosurgery Publishing Group; 2002;97: 1054–1061. doi: 10.3171/jns.2002.97.5.1054 12450026 10.3171/jns.2002.97.5.1054

[43] Rangel-Castilla L, Lara LR, Gopinath S, Swank PR, Valadka A, Robertson C. Cerebral hemodynamic effects of acute hyperoxia and hyperventilation after severe traumatic brain injury. J Neurotrauma. Mary Ann Liebert, Inc. 140 Huguenot Street, 3rd Floor New Rochelle, NY 10801 USA; 2010;27: 1853–63. doi: 10.1089/neu.2010.1339 20684672 10.1089/neu.2010.1339 PMC2953927

[44] Sviri GE, Aaslid R, Douville CM, Moore A, Newell DW. Time course for autoregulation recovery following severe traumatic brain injury. J Neurosurg. American Association of Neurological Surgeons; 2009;111: 695–700. doi: 10.3171/2008.10.17686 19392589 10.3171/2008.10.17686

[45] Schramm P, Klein KU, Pape M, Berres M, Werner C, Kochs E, et al Serial measurement of static and dynamic cerebrovascular autoregulation after brain injury. J Neurosurg Anesthesiol. 2011;23: 41–4. doi: 10.1097/ANA.0b013e3181f35854 21252706 10.1097/ANA.0b013e3181f35854

[46] Salam A, Smina M, Gada P, Tilluckdharry L, Upadya A, Amoateng-Adjepong Y, et al The effect of arterial blood gas values on extubation decisions. Respir Care. Respiratory Care; 2003;48: 1033–7. 14585115

[47] Ogawa Y, Iwasaki K, Aoki K, Gokan D, Hirose N, Kato J, et al The different effects of midazolam and propofol sedation on dynamic cerebral autoregulation. Anesth Analg. 2010;111: 1279–1284. doi: 10.1213/ANE.0b013e3181f42fc0 20881283 10.1213/ANE.0b013e3181f42fc0

[48] Lavinio A, Timofeev I, Nortje J, Outtrim J, Smielewski P, Gupta A, et al Cerebrovascular reactivity during hypothermia and rewarming. Br J Anaesth. 2007;99: 237–244. doi: 10.1093/bja/aem118 17510046 10.1093/bja/aem118

[49] Oertel M, Boscardin WJ, Obrist WD, Glenn TC, McArthur DL, Gravori T, et al Posttraumatic vasospasm: the epidemiology, severity, and time course of an underestimated phenomenon: a prospective study performed in 299 patients. J Neurosurg. Journal of Neurosurgery Publishing Group; 2005;103: 812–824. doi: 10.3171/jns.2005.103.5.0812 16304984 10.3171/jns.2005.103.5.0812

[50] Donnelly J, Czosnyka M, Sudhan N, Varsos G V., Nasr N, Jalloh I, et al Increased Blood Glucose is Related to Disturbed Cerebrovascular Pressure Reactivity After Traumatic Brain Injury. Neurocrit Care. 2014;22: 20–25. doi: 10.1007/s12028-014-0042-4 25124103 10.1007/s12028-014-0042-4

[51] Dias C, Gaio AR, Monteiro E, Barbosa S, Cerejo A, Donnelly J, et al Kidney-Brain Link in Traumatic Brain Injury Patients? A preliminary report. Neurocrit Care. 2015;22: 192–201. doi: 10.1007/s12028-014-0045-1 25273515 10.1007/s12028-014-0045-1

[52] Sekhon MS, Griesdale DE, Czosnyka M, Donnelly J, Liu X, Aries MJ, et al The Effect of Red Blood Cell Transfusion on Cerebral Autoregulation in Patients with Severe Traumatic Brain Injury. Neurocrit Care. 2015;23: 210–6. doi: 10.1007/s12028-015-0141-x 25894454 10.1007/s12028-015-0141-x

[53] Pfister D, Siegemund M, Dell-Kuster S, Smielewski P, Rüegg S, Strebel SP, et al Cerebral perfusion in sepsis-associated delirium. Crit Care. 2008;12: R63 doi: 10.1186/cc6891 18457586 10.1186/cc6891 PMC2481444

[54] Taccone FS, Castanares-Zapatero D, Peres-Bota D, Vincent J-L, Berre’ J, Melot C. Cerebral autoregulation is influenced by carbon dioxide levels in patients with septic shock. Neurocrit Care. 2010;12: 35–42. doi: 10.1007/s12028-009-9289-6 19806473 10.1007/s12028-009-9289-6

[55] Ameloot K, Genbrugge C, Meex I, Jans F, Boer W, Vander Laenen M, et al An observational near-infrared spectroscopy study on cerebral autoregulation in post-cardiac arrest patients: Time to drop “one-size-fits-all” hemodynamic targets? Resuscitation. 2015;90: 121–126. doi: 10.1016/j.resuscitation.2015.03.001 25769511 10.1016/j.resuscitation.2015.03.001

[56] Fanelli V, Mazzeo A, Battaglini I, Caccia S, Boffini M, Ricci D, et al CEREBRAL AUTOREGULATION IN PATIENTS TREATED WITH V-VECMO FOR SEVERE ARDS. Intensive Care Med Exp. Springer International Publishing; 2015;3: A509 doi: 10.1186/2197-425X-3-S1-A509

[57] Sykora M, Czosnyka M, Liu X, Donnelly J, Nasr N, Diedler J, et al Autonomic Impairment in Severe Traumatic Brain Injury: A Multimodal Neuromonitoring Study. Crit Care Med. 2016;Publish Ah: 1–9. doi: 10.1097/CCM.0000000000001624 26968025 10.1097/CCM.0000000000001624

[58] Robertson CS, Narayan RK, Contant CF, Grossman RG, Gokaslan ZL, Pahwa R, et al Clinical experience with a continuous monitor of intracranial compliance. J Neurosurg. Journal of Neurosurgery Publishing Group; 1989;71: 673–680. doi: 10.3171/jns.1989.71.5.0673 2681566 10.3171/jns.1989.71.5.0673

[59] Timofeev I, Czosnyka M, Nortje J, Smielewski P, Kirkpatrick P, Gupta A, et al Effect of decompressive craniectomy on intracranial pressure and cerebrospinal compensation following traumatic brain injury. J Neurosurg. 2008;108: 66–73. doi: 10.3171/JNS/2008/108/01/0066 18173312 10.3171/JNS/2008/108/01/0066

[60] Werner C, Engelhard K. Pathophysiology of traumatic brain injury. Br J Anaesth. Oxford University Press; 2007;99: 4–9. doi: 10.1093/bja/aem131 17573392 10.1093/bja/aem131

[61] Golding EM, Robertson CS, Bryan RM. The consequences of traumatic brain injury on cerebral blood flow and autoregulation: a review. Clin Exp Hypertens. 1999;21: 299–332. 10369378 10.3109/10641969909068668

