# Supplementary material for: Temporal profile of intracranial pressure and cerebrovascular reactivity in severe traumatic brain injury and association with fatal outcome: An observational study
Source: PLoS Med. 2017 Jul 25;14(7):e1002353. doi: 10.1371/journal.pmed.1002353 (PMC5526498; doi:10.1371/journal.pmed.1002353)
Supplement: S1 Appendix — (PDF) [file pmed.1002353.s001.pdf]

S1 Appendix: Transparent reporting of a multivariable prediction model for individual prognosis or diagnosis (TRIPOD) Checklist.

Manuscript: Temporal profile of intracranial pressure and cerebrovascular reactivity in severe traumatic brain injury and association with fatal outcome: an observational study.

Authors: Hadie Adams[1], Joseph Donnelly[1], Marek Czosnyka[1,2], Angelos G Kolias[1], Adel Helmy[1], David K Menon[3], Peter Smielewski[1*], Peter J Hutchinson[1*]

1. Division of Neurosurgery, Depart. of Clinical Neuroscience, Box 167, Addenbrooke's Hospital, University of Cambridge, Cambridge, UK
2. Institute of Electronic Systems, Warsaw University of technology, Poland

Department of Anaesthesia, Addenbrooke's Hospital, University of Cambridge,

3. Department of Anaesthesia, Addenbrooke's Hospital, University of Cambridge, Cambridge, UK

TRIPOD Checklist: Prediction Model Development

Section/Topic	Item	Checklist Item	Section
**Title and abstract**			
Title	1	Identify the study as developing and/or validating a multivariable prediction model, the target population, and the outcome to be predicted.	Title page
Abstract	2	Provide a summary of objectives, study design, setting, participants, sample size, predictors, outcome, statistical analysis, results, and conclusions.	Abstract
**Introduction**			
Background and objectives	3a	Explain the medical context (including whether diagnostic or prognostic) and rationale for developing or validating the multivariable prediction model, including references to existing models.	Introduction paragraph 4 & 5
	3b	Specify the objectives, including whether the study describes the development or validation of the model or both.	Introduction paragraph 5
**Methods**			
Source of data	4a	Describe the study design or source of data (e.g., randomized trial, cohort, or registry data), separately for the development and validation data sets, if applicable.	Methods paragraph 1
	4b	Specify the key study dates, including start of accrual; end of accrual; and, if applicable, end of follow-up.	Methods paragraph 1
Participants	5a	Specify key elements of the study setting (e.g., primary care, secondary care, general population) including number and location of centres.	Methods paragraph 1
	5b	Describe eligibility criteria for participants.	Methods paragraph 1
	5c	Give details of treatments received, if relevant.	Methods paragraph 1 & 3
Outcome	6a	Clearly define the outcome that is predicted by the prediction model, including how and when assessed.	Methods paragraph 3-5
	6b	Report any actions to blind assessment of the outcome to be predicted.	N/A
Predictors	7a	Clearly define all predictors used in developing or validating the multivariable prediction model, including how and when they were measured.	Methods paragraph 3
	7b	Report any actions to blind assessment of predictors for the outcome and other predictors.	N/A
Sample size	8	Explain how the study size was arrived at.	Methods paragraph 1
Missing data	9	Describe how missing data were handled (e.g., complete-case analysis, single imputation, multiple imputation) with details of any imputation method.	Methods paragraph 8 & 9
Statistical analysis methods	10a	Describe how predictors were handled in the analyses.	Methods paragraph 10-13
	10b	Specify type of model, all model-building procedures (including any predictor selection), and method for internal validation.	Methods paragraph 11-13
	10d	Specify all measures used to assess model performance and, if relevant, to compare multiple models.	Methods paragraph 13
Risk groups	11	Provide details on how risk groups were created, if done.	N/A
**Results**			
Participants	13a	Describe the flow of participants through the study, including the number of participants with and without the outcome and, if applicable, a summary of the follow-up time. A diagram may be helpful.	Results paragraph 1, Table 1 & 2
	13b	Describe the characteristics of the participants (basic demographics, clinical features, available predictors), including the number of participants with missing data for predictors and outcome.	Results paragraph 1, Table 1 & 2
Model development	14a	Specify the number of participants and outcome events in each analysis.	Results paragraph 1, Table 2, Figure 2
	14b	If done, report the unadjusted association between each candidate predictor and outcome.	N/A
Model specification	15a	Present the full prediction model to allow predictions for individuals (i.e., all regression coefficients, and model intercept or baseline survival at a given time point).	Results paragraph 5, Table 3
	15b	Explain how to the use the prediction model.	N/A
Model performance	16	Report performance measures (with CIs) for the prediction model.	Results paragraph 6, Figure 3, S4 Support. Inf.
**Discussion**			

Limitations	18	Discuss any limitations of the study (such as nonrepresentative sample, few events per predictor, missing data).	Discussion paragraph 11-13
Interpretation	19b	Give an overall interpretation of the results, considering objectives, limitations, and results from similar studies, and other relevant evidence.	Discussion Paragraph 1-10
Implications	20	Discuss the potential clinical use of the model and implications for future research.	Discussion Paragraph 8-10, 14
**Other information**			
Supplementary information	21	Provide information about the availability of supplementary resources, such as study protocol, Web calculator, and data sets.	See data availability statement
Funding	22	Give the source of funding and the role of the funders for the present study.	See funding statement

We recommend using the TRIPOD Checklist in conjunction with the TRIPOD Explanation and Elaboration document.
