# Supplementary material for: Temporal profile of intracranial pressure and cerebrovascular reactivity in severe traumatic brain injury and association with fatal outcome: An observational study
Source: PLoS Med. 2017 Jul 25;14(7):e1002353. doi: 10.1371/journal.pmed.1002353 (PMC5526498; doi:10.1371/journal.pmed.1002353)
Supplement: S2 Appendix — (PDF) [file pmed.1002353.s002.pdf]

S2 Appendix: Original analysis plan and modifications following comments from editors and reviewers

Manuscript: Temporal profile of intracranial pressure and cerebrovascular reactivity in severe traumatic brain injury and association with fatal outcome: an observational study.

Authors: Hadie Adams[1], Joseph Donnelly[1], Marek Czosnyka[1,2], Angelos G Kolias[1], Adel Helmy[1], David K Menon[3], Peter Smielewski[1*], Peter J Hutchinson[1*]

1. Division of Neurosurgery, Depart. of Clinical Neuroscience, Box 167, Addenbrooke's Hospital, University of Cambridge, Cambridge, UK
2. Institute of Electronic Systems, Warsaw University of technology, Poland

Department of Anaesthesia, Addenbrooke's Hospital, University of Cambridge,

3. Department of Anaesthesia, Addenbrooke's Hospital, University of Cambridge, Cambridge, UK

Main objective: To investigate the associations of the temporal profile of ICP and PRx to TBI specific mortality.

Participants: All sTBI participants with high resolution ICP and ABP recordings (n=601) will be included.

- For the outcomes analyses of TBI specific mortality, patients dying from non-neurological cases and non-functional survivors will be excluded, leaving 556 patients in the main analyses.

Exposure: Periods of raised ICPs and cerebrovascular dysregulation are captured with computerised continues monitoring.

Outcomes: Time and cause of death and contributing factors to mortality were determined by review of hospital records or by acquisition of death certificate or autopsy reports.

- Fatal outcome and functional survivors:
  - o Fatal outcome: Cause-specific mortality will be categorised in two groups: those that died due to neurological causes (non-survivable TBI or brain death) and patients with a fatal outcome who died from non-neurological causes (i.e. respiratory failure, sepsis, myocardial infarction).
  - o Functional Survivors: Survivors are defined as those alive at six months post-injury. Functional survivors are defined as those surviving the injury with severe disability, moderate disability, or good recovery at six months post-injury. Hence, patients in a vegetative state are excluded from further outcome analysis, as those are non-functional survivors, and are known to be atypical in their characteristics.

Statistical analyses: Mixed effects models.

- To assess the difference in brain physiological parameter and their trajectories between those with a fatal outcome and functional survivors, a linear mixed-effects model (LMEM) with a between-subjects factor (group: fatal vs. non-fatal), a within-subject factor (time: T_{24} to T_{240}) and the interaction between these two with patient ID as a random effect. Best model fit will be based on the Akaike Information Criterion.

- A generalized linear mixed model (GLMM) will be used to examine the effect of ICP and PRx on the probability [odds ratio (OR)] of having fatal outcome over time, using repeated

measures of these parameters over the first 240 hours post-injury. The model also included the same fixed and random effects and interactions term as the LMEM model.

- To test how well ICP and PRx could distinguish between fatal outcome and functional survivors during different time points (T_{24}-T_{240}), the area under the receiver operating characteristic curve (ROC AUC) will be calculated and compared.

Covariates: Selected based on literature and a priori knowledge about our data (resulting from previous work examining the association of ICP, PRx and outcome):

- Age as a continues variables
- Sex
- Best-pre-intubation GCS (3-15) as an ordinal scale
- Primary injury type (Diffuse vs. Mass lesion)
- Surgical interventions (none, craniotomy, primary DC, and secondary DC)
- DC as an adaptive intervention parameter.

Sensitivity analyses: In order to examine the potential influence of selection bias, it is important to check if the cohort is not 'expiring' because of mortality taken place at particular time-points. The peak incidence of death at any time point and the proportionality of the number of deaths to the survivors will be tested at every time point (T_{24} - T_{240}).

Subgroup analyses: In this study no subgroup analyses are specified. However, several studies have been planned to identify potential effect modifiers of the time-course of ICP and PRx.

Modification based on the comments from editors and reviewers: Following the suggestion of reviewers, we performed the mixed effect model analyses, additionally adjusting for the year of admission.
