# Supplementary material for: Temporal profile of intracranial pressure and cerebrovascular reactivity in severe traumatic brain injury and association with fatal outcome: An observational study
Source: PLoS Med. 2017 Jul 25;14(7):e1002353. doi: 10.1371/journal.pmed.1002353 (PMC5526498; doi:10.1371/journal.pmed.1002353)
Supplement: S1 Supporting Information — (PDF) [file pmed.1002353.s003.pdf]

Supporting Information 1: Traumatic Brain Injury ICP/CPP Algorithm at the Neurosciences and Trauma Critical Care Unit, Addenbrooke's Hospital, Cambridge, UK

Manuscript: Temporal profile of intracranial pressure and cerebrovascular reactivity in severe traumatic brain injury and association with fatal outcome: an observational study.

Authors: Hadie Adams[1], Joseph Donnelly[1], Marek Czosnyka[1,2], Angelos G Kolias[1], Adel Helmy[1], David K Menon[3], Peter Smielewski[1*], Peter J Hutchinson[1*]

1. Division of Neurosurgery, Depart. of Clinical Neuroscience, Box 167, Addenbrooke's Hospital, University of Cambridge, Cambridge, UK
2. Institute of Electronic Systems, Warsaw University of technology, Poland
Department of Anaesthesia, Addenbrooke's Hospital, University of Cambridge,
3. Department of Anaesthesia, Addenbrooke's Hospital, University of Cambridge, Cambridge, UK

Traumatic Brain Injury ICP/CPP Algorithm

Patients with traumatic brain injury admitted to NCCU are managed according to this protocol. Each step of the protocol must be preceded by thorough check of the position and accuracy of all intracranial monitoring. Surgical referral for evacuation of significant space occupying lesions (SOL) is mandatory before escalating medical treatment. Consider EVD insertion before escalating medical treatment. All patients must have the following within 4h of admission to NCCU:

1) Invasive arterial (transducer at tragus) and central venous catheter
2) ICP monitoring
3) Cerebral microdialysis catheter and PbO_2 probe
4) ICM+

Initial target CPP of 65mmHg (CPP > 55mmHg may be acceptable). Autoregulation parameters and brain biochemistry are used to individualise targets.

Stage 1

Adequate resuscitation, ensure euvolemia, add inotropes and vasopressors if needed. FICE echo.
Elevate head to 30° unless clinically contraindicated (otherwise 10-15°)
SpO_2 >94%; $PaCO_2$ 4.5 – 5.0kPa
CPP 55 - 65 mmHg – transducer at tragus.
Hb >90
Blood glucose 4 - 10mmol/l; (brain glucose >0.5mmol/l)
Multimodal monitoring: PRx <0.2 (use CPP_{opt}); PbO_2 >15; LPR <25
Targeted temperature management to 37°C (regular paracetamol + cooling);
Propofol 2 - 5mg/kg/hr; fentanyl 1 - 4 micrograms/kg/hr; consider atracurium 0.5mg/kg/hr.
AED if seizures suspected (first line – levetiracetam – 1g IV OD; second line – phenytoin 20mg/kg IV loading then 100 mg IV TDS); EEG

→ If ICP >20 mmHg escalate to STAGE 2, consider rescan and evacuation of SOL

Stage 2

Drain CSF – EVD at 5 - 10 cmH_2O
5% NaCl 2ml/kg IV (until plasma Na >160 mmol/l or plasma osmolality ≈320 mOsm/Kg)
Mild hypothermia, temp ≈35°C, daily lipids, ECG and CK if still on propofol

→ If ICP >20 mmHg escalate to STAGE 3, consider rescan and evacuation of SOL

Stage 3

Trial of CPP >70mmHg
Advanced haemodynamic monitoring – further optimise cardiovascular function
AED if not already initiated
Daily lipids, ECG and CK if still on propofol
On consultant approval only: $PaCO_2$ to ≈4.0 kPa and monitor brain oxygenation

→ If ICP >25 mmHg escalate to STAGE 4, consider rescan and evacuation of SOL

Stage 4 (NCCU consultant approval only)

Moderate hypothermia to 32 – 34°C. Change to midazolam (± max 2mg/kg/hr propofol)
Daily lipids, ECG and CK

→ If ICP >25 mmHg escalate to STAGE 5, consider rescan and evacuation of SOL

Stage 5 (NCCU consultant approval only)

Add IV anaesthetic (e.g. propofol 1mg/kg IV stat) - maintain CPP
If ICP and CPP improve, start thiopental: 250mg boluses up to 3 - 5g, then 3 - 8mg/kg/hr
Burst suppression ratio »50%

→ Consider decompressive craniectomy (mandatory NCCU consultant discussion)
