# Supplementary material for: Temporal profile of intracranial pressure and cerebrovascular reactivity in severe traumatic brain injury and association with fatal outcome: An observational study
Source: PLoS Med. 2017 Jul 25;14(7):e1002353. doi: 10.1371/journal.pmed.1002353 (PMC5526498; doi:10.1371/journal.pmed.1002353)
Supplement: S2 Supporting Information — (PDF) [file pmed.1002353.s004.pdf]

S2 Supporting Information: Patient data excluded due to low coverage of 24-hour epoch.

Manuscript: Temporal profile of intracranial pressure and cerebrovascular reactivity in severe traumatic brain injury and association with fatal outcome: an observational study.

Authors: Hadie Adams[1], Joseph Donnelly[1], Marek Czosnyka[1,2], Angelos G Kolias[1], Adel Helmy[1], David K Menon[3], Peter Smielewski[1*], Peter J Hutchinson[1*]

1. Division of Neurosurgery, Depart. of Clinical Neuroscience, Box 167, Addenbrooke's Hospital, University of Cambridge, Cambridge, UK
2. Institute of Electronic Systems, Warsaw University of technology, Poland
Department of Anaesthesia, Addenbrooke's Hospital, University of Cambridge,
3. Department of Anaesthesia, Addenbrooke's Hospital, University of Cambridge, Cambridge, UK

**Table A.** Recording time of ICP of included and excluded patients epochs (due to <50% recording time available per 24 hour epoch) of 556 sTBI patients for each 24h epoch.				
	**Excluded Patients Epochs**		**Included Patients Epochs**	
**24h epoch**	**N**	**Median ICP (IQR) recording time in hours**	**N**	**Median ICP (IQR) recording time in hours**
1 (0-24h)	116	4.08 (3.04)	43	16.80 (5.95)
2 (24-48h)	86	5.58 (3.65)	254	19.95 (7.29)
3 (48-72h)	74	4.73 (4.45)	346	22.13 (6.23)
4 (72-96h)	50	4.57 (4.87)	375	22.33 (5.20)
5 (96-120h)	46	4.88 (3.73)	357	22.98 (4.64)
6 (120-144h)	47	4.83 (4.05)	327	22.78 (4.65)
7 (144-168h)	34	4.32 (4.19)	297	23.07 (5.52)
8 (168-192h)	27	4.51 (3.80)	254	23.52 (4.53)
9 (192-216h)	34	4.38 (3.91)	216	22.82 (5.11)
10 (216-240h)	33	4.05 (3.47)	182	23.21 (4.83)

**Table B.** Mean ICP values of included and excluded patients epochs (due to <50% recording time available per 24 hour epoch) of 556 sTBI patients for each 24h epoch.			
	**Excluded Patients Epochs**	**Included Patients Epochs**	
**24h epoch**	**Mean ICP (SD)**	**Mean ICP (SD)**	**Intergroup Differences**
1 (0-24h)	17.6 (9.2)	15.3 (7.3)	-
2 (24-48h)	17.6 (11.5)	16.8 (7.1)	-
3 (48-72h)	18.1 (11.6)	16.1 (6.7)	-
4 (72-96h)	17.2 (12.6)	15.6 (6.5)	$p=0.48$
5 (96-120h)	17.7 (14.2)	15.7 (8.1)	-
6 (120-144h)	14.4 (8.4)	16.3 (8.8)	-
7 (144-168h)	14.2 (7.9)	16.6 (9.7)	-
8 (168-192h)	19.3 (19.8)	16.8 (9.4)	-
9 (192-216h)	15.3 (11.7)	16.4 (9.2)	-
10 (216-240h)	22.4 (16.9)	15.6 (8.3)	$p<0.001$

**Table C.** Recording time of PRx of included and excluded patients epochs (due to <50% recording time available per 24 hour epoch) of 556 sTBI patients for each 24h epoch.				
	**Excluded Patients Epochs**		**Included Patients Epochs**	
**24h epoch**	**N**	**Median PRx (IQR) recording time in hours**	**N**	**Median PRx (IQR) recording time in hours**
1 (0-24h)	115	3.80 (3.00)	36	16.59 (5.60)
2 (24-48h)	84	5.00 (3.67)	243	19.10 (7.81)
3 (48-72h)	77	4.33 (4.83)	326	21.22 (6.82)
4 (72-96h)	47	4.50 (3.90)	356	21.45 (6.17)
5 (96-120h)	47	4.72 (3.76)	335	22.07 (5.35)
6 (120-144h)	53	4.59 (4.28)	302	22.00 (6.67)
7 (144-168h)	32	3.75 (4.45)	276	22.36 (5.82)
8 (168-192h)	29	5.17 (4.25)	235	22.68 (4.82)
9 (192-216h)	34	4.33 (4.02)	200	22.02 (5.10)
10 (216-240h)	31	3.97 (3.37)	170	22.13 (6.10)

**Table D.** Mean PRx values of included and excluded patients epochs (due to <50% recording time available per 24 hour epoch) of 556 sTBI patients for each 24h epoch.

	Excluded Patients Epochs	Included Patients Epochs	
**24h epoch**	**Mean PRx (SD)**	**Mean PRx (SD)**	**Intergroup Differences**
1 (0-24h)	0.09 (0.22)	0.13 (0.20)	-
2 (24-48h)	0.04 (0.25)	0.04 (0.23)	-
3 (48-72h)	0.05 (0.26)	0.02 (0.24)	-
4 (72-96h)	0.09 (0.24)	-0.01 (0.21)	p=0.002
5 (96-120h)	0.15 (0.27)	0.01 (0.20)	p<0.001
6 (120-144h)	0.10 (0.22)	0.04 (0.21)	-
7 (144-168h)	0.08 (0.24)	0.06 (0.20)	-
8 (168-192h)	0.13 (0.25)	0.09 (0.20)	-
9 (192-216h)	0.17 (0.21)	0.10 (0.21)	-
10 (216-240h)	0.20 (0.30)	0.09 (0.19)	p=0.006

Table E. Sample size of included patients 556 sTBI patients for each 24h epoch stratified by fatal outcome (due to non-survivable TBI or brain death) and functional survivors (ranging from severe disability to good recovery) at 6 months post-injury.

	ICP			PRx		
**24h epoch**	**Functional Survivors N**	**Fatal Outcome N**	**Total N**	**Functional Survivors N**	**Fatal Outcome N**	**Total N**
1 (0-24h)	34	9	43	28	8	36
2 (24-48h)	212	42	254	204	39	243
3 (48-72h)	296	50	346	275	51	326
4 (72-96h)	323	52	375	306	50	356
5 (96-120h)	304	53	357	283	52	335
6 (120-144h)	270	57	327	246	56	302
7 (144-168h)	243	54	297	224	52	276
8 (168-192h)	209	45	254	191	44	235
9 (192-216h)	178	38	216	164	36	200
10 (216-240h)	154	28	182	144	26	170

Table F. Sample size of excluded patients epochs (due to <50% recording time available per 24 hour epoch) for each 24h epoch of 556 sTBI patients stratified by fatal outcome (due to non-survivable TBI or brain death) and functional survivors (ranging from severe disability to good recovery) at 6 months post-injury.

	ICP			PRx		
**24h epoch**	**Functional Survivors N**	**Fatal Outcome N**	**Total N**	**Functional Survivors N**	**Fatal Outcome N**	**Total N**
1 (0-24h)	97	19	116	97	18	115
2 (24-48h)	71	15	86	67	17	84
3 (48-72h)	61	13	74	66	11	77
4 (72-96h)	37	13	50	34	13	47
5 (96-120h)	36	10	46	38	9	47
6 (120-144h)	43	4	47	50	3	53
7 (144-168h)	33	1	34	31	1	32
8 (168-192h)	22	5	27	24	5	29
9 (192-216h)	31	3	34	30	4	34
10 (216-240h)	24	9	33	22	9	31
