# Supplementary material for: Temporal profile of intracranial pressure and cerebrovascular reactivity in severe traumatic brain injury and association with fatal outcome: An observational study
Source: PLoS Med. 2017 Jul 25;14(7):e1002353. doi: 10.1371/journal.pmed.1002353 (PMC5526498; doi:10.1371/journal.pmed.1002353)
Supplement: S3 Supporting Information — (PDF) [file pmed.1002353.s005.pdf]

S3 Supporting Information: Tabulated heatmaps from Figure 1A-D.

Manuscript: Temporal profile of intracranial pressure and cerebrovascular reactivity in severe traumatic brain injury and association with fatal outcome: an observational study.

Authors: Hadie Adams[1], Joseph Donnelly[1], Marek Czosnyka[1,2], Angelos G Kolias[1], Adel Helmy[1], David K Menon[3], Peter Smielewski[1*], Peter J Hutchinson[1*]

1. Division of Neurosurgery, Depart. of Clinical Neuroscience, Box 167, Addenbrooke's Hospital, University of Cambridge, Cambridge, UK
2. Institute of Electronic Systems, Warsaw University of technology, Poland
Department of Anaesthesia, Addenbrooke's Hospital, University of Cambridge,
3. Department of Anaesthesia, Addenbrooke's Hospital, University of Cambridge, Cambridge, UK

**Table A.** Tabulated values of Fig 1A. Mean ICP of 601 sTBI patients stratified by Glasgow Outcome Scale at 6 months post-injury, and further divided by cause of death.						
**24h epoch**	**Death (non-neurological causes)**	**Death (neurological cause)**	**Vegetative State**	**Severe Disability**	**Moderate Disability**	**Good Recovery**
1 (0-24h)	22.3	18.7	14.6	16.7	14.8	14.5
2 (24-48h)	14.2	20.6	14.5	17.4	15.0	15.2
3 (48-72h)	13.1	19.4	12.7	16.2	15.8	14.9
4 (72-96h)	12.0	19.6	11.7	15.1	15.1	14.5
5 (96-120h)	12.8	18.2	13.0	15.4	15.0	15.1
6 (120-144h)	12.8	18.9	14.7	15.5	16.4	15.1
7 (144-168h)	12.3	19.0	15.8	16.3	16.7	15.9
8 (168-192h)	12.3	19.1	15.9	16.0	16.7	16.0
9 (192-216h)	10.8	19.4	16.0	15.7	15.8	15.8
10 (216-240h)	10.9	17.7	17.8	15.1	15.3	16.7

**Table B.** Tabulated values of Fig 1B. Mean PRx of 601 sTBI patients stratified by Glasgow Outcome Scale at 6 months post-injury, and further divided by cause of death.						
**24h epoch**	**Death (non-neurological causes)**	**Death (neurological cause)**	**Vegetative State**	**Severe Disability**	**Moderate Disability**	**Good Recovery**
1 (0-24h)	0.06	0.36	0.11	0.09	0.04	-0.02
2 (24-48h)	0.13	0.24	0.11	0.01	0.03	-0.04
3 (48-72h)	0.10	0.16	0.09	0.00	-0.02	-0.02
4 (72-96h)	0.10	0.09	0.08	-0.01	-0.02	-0.05
5 (96-120h)	0.12	0.09	-0.11	0.02	-0.02	-0.02
6 (120-144h)	0.17	0.10	-0.02	0.03	0.00	0.01
7 (144-168h)	0.23	0.10	-0.04	0.07	0.03	0.03
8 (168-192h)	0.22	0.13	-0.02	0.10	0.08	0.02
9 (192-216h)	0.22	0.16	0.04	0.11	0.06	0.04
10 (216-240h)	0.27	0.12	0.07	0.09	0.08	0.07

**Table C.** Tabulated values of Fig 1C. Mean % time ICP > 25 mm Hg of 601 sTBI patients stratified by Glasgow Outcome Scale at 6 months post-injury, and further divided by cause of death.						
**24h epoch**	**Death (non-neurological causes)**	**Death (neurological cause)**	**Vegetative State**	**Severe Disability**	**Moderate Disability**	**Good Recovery**
1 (0-24h)	20.9	24.4	21.0	11.0	9.5	8.1
2 (24-48h)	6.4	25.5	10.3	11.0	7.6	7.3
3 (48-72h)	5.7	21.0	4.1	9.5	8.8	5.4
4 (72-96h)	5.1	19.3	1.9	7.9	6.2	6.2
5 (96-120h)	10.3	18.4	4.2	8.1	6.8	8.3
6 (120-144h)	4.6	23.3	3.9	8.6	6.5	11.2
7 (144-168h)	5.2	20.8	3.5	9.1	7.7	13.0
8 (168-192h)	5.6	20.9	3.8	10.1	8.8	14.1
9 (192-216h)	2.2	23.4	11.1	9.4	10.4	11.5
10 (216-240h)	0.7	16.6	16.4	8.9	13.2	11.6

**Table D.** Tabulated values of Fig 1D. Mean % time PRx > 0.25 a.u. of 601 sTBI patients stratified by Glasgow Outcome Scale at 6 months post-injury, and further divided by cause of death.						
**24h epoch**	**Death (non-neurological causes)**	**Death (neurological cause)**	**Vegetative State**	**Severe Disability**	**Moderate Disability**	**Good Recovery**
1 (0-24h)	10.5	69.1	37.5	37.5	29.8	25.5
2 (24-48h)	42.5	53.5	34.2	30.3	31.1	25.3
3 (48-72h)	38.6	46.5	34.3	28.3	26.6	24.2
4 (72-96h)	39.6	39.5	38.0	28.0	26.1	24.2
5 (96-120h)	40.5	39.0	20.9	31.2	28.1	26.4
6 (120-144h)	45.9	39.7	26.5	32.9	33.2	29.7
7 (144-168h)	52.7	40.7	27.3	37.3	33.2	32.3

8 (168-192h)	51.9	43.6	28.1	40.5	37.2	31.2
9 (192-216h)	51.1	47.3	34.4	41.9	36.4	35.9
10 (216-240h)	55.9	43.5	36.7	38.7	38.0	39.2
