# Supplementary material for: Temporal profile of intracranial pressure and cerebrovascular reactivity in severe traumatic brain injury and association with fatal outcome: An observational study
Source: PLoS Med. 2017 Jul 25;14(7):e1002353. doi: 10.1371/journal.pmed.1002353 (PMC5526498; doi:10.1371/journal.pmed.1002353)
Supplement: S4 Supporting Information — (PDF) [file pmed.1002353.s006.pdf]

S4 Supporting Information: Tabulated data from Figure 3 (time-dependent AUC-ROC).

Manuscript: Temporal profile of intracranial pressure and cerebrovascular reactivity in severe traumatic brain injury and association with fatal outcome: an observational study.

Authors: Hadie Adams[1], Joseph Donnelly[1], Marek Czosnyka[1,2], Angelos G Kolias[1], Adel Helmy[1], David K Menon[3], Peter Smielewski[1*], Peter J Hutchinson[1*]

1. Division of Neurosurgery, Depart. of Clinical Neuroscience, Box 167, Addenbrooke's Hospital, University of Cambridge, Cambridge, UK
2. Institute of Electronic Systems, Warsaw University of technology, Poland

Department of Anaesthesia, Addenbrooke's Hospital, University of Cambridge,
3. Department of Anaesthesia, Addenbrooke's Hospital, University of Cambridge, Cambridge, UK

Table A. Tabulated values of Fig 3. Time-dependent ROC curve analysis for prediction of fatal outcome due to neurological causes. The evolution of AUC-ROC of 3 different models over time have been plotted: GLMM Model 1 – static (constant) variables with patient, injury and treatment characteristics. GLMM Model 2 – repeated measures of ICP including static variables from Model 1. GLMM Model 3 – repeated measures of PRx including static variables from Model 1. At each time-point GLMM Models 2 & 3 are using repeated measures of the dynamic variables (ICP and PRx) from baseline to the respective time point. Due to the limited sample size for T24, data has not calculated and plotted for this time-point separately. Abbreviations: ROC = receiver operating characteristic; AUC = area under the ROC curve; GLMM = Generalized Linear Mixed Model.

**Data epochs**	**GLMM Model 1 Constant Variables AUC-ROC (95%)**		**GLMM Model 2 Constant + ICP AUC-ROC (95%)**		**GLMM Model 3 Constant + PRx AUC-ROC (95%)**	
0-48h	0.69	(0.65-0.73)	0.80	(0.74-0.87)	0.86	(0.80-0.91)
0-72h	0.69	(0.65-0.72)	0.80	(0.76-0.84)	0.81	(0.76-0.86)
0-96h	0.69	(0.66-0.72)	0.79	(0.76-0.83)	0.79	(0.75-0.83)
0-120h	0.69	(0.66-0.71)	0.78	(0.74-0.81)	0.77	(0.73-0.80)
0-144h	0.69	(0.66-0.71)	0.77	(0.74-0.80)	0.76	(0.73-0.79)
0-168h	0.69	(0.67-0.71)	0.77	(0.74-0.79)	0.76	(0.73-0.79)
0-192h	0.69	(0.67-0.71)	0.76	(0.74-0.79)	0.75	(0.72-0.78)
0-216h	0.69	(0.67-0.71)	0.76	(0.73-0.78)	0.75	(0.72-0.77)
0-240h	0.69	(0.67-0.71)	0.75	(0.73-0.78)	0.74	(0.72-0.77)
